# CRISPR-mediated gap-based replacement enables site-specific DNA lesion insertion into long DNA and nucleosomes

**DOI:** 10.1093/nar/gkag965

**Published:** 2026-10-13

**Authors:** Youngseo Kim, Soyeong An, Jimin Choi, Moonjung Choi, Gyeongjin Lee, Kei-ichi Takata, Roopesh Anand, Kyungjae Myung, Ja Yil Lee

**Affiliations:** Department of biological sciences, Ulsan National Institute of Science and Technology, Ulsan 44919, Republic of Korea; Department of biological sciences, Ulsan National Institute of Science and Technology, Ulsan 44919, Republic of Korea; Department of biological sciences, Ulsan National Institute of Science and Technology, Ulsan 44919, Republic of Korea; Institute for Basic Science Center for Genomic Integrity, Ulsan 44919, Republic of Korea; Department of biological sciences, Ulsan National Institute of Science and Technology, Ulsan 44919, Republic of Korea; Institute for Basic Science Center for Genomic Integrity, Ulsan 44919, Republic of Korea; Department of biomedical engineering, Ulsan National Institute of Science and Technology, Ulsan 44919, Republic of Korea; Institute of Molecular Biology (IMB), Mainz 55128, Germany; Institute for Basic Science Center for Genomic Integrity, Ulsan 44919, Republic of Korea; Department of biomedical engineering, Ulsan National Institute of Science and Technology, Ulsan 44919, Republic of Korea; Department of biological sciences, Ulsan National Institute of Science and Technology, Ulsan 44919, Republic of Korea; Institute for Basic Science Center for Genomic Integrity, Ulsan 44919, Republic of Korea

## Abstract

Defined DNA lesions are essential substrates for dissecting DNA repair mechanisms, yet existing approaches remain limited in positional flexibility, lesion diversity, efficiency, or compatibility with long and chromatinized DNA. Here, we present CGRID (CRISPR-mediated Gap-based Replacement for Insertion of DNA damage), a programmable strategy for site-specific installation of DNA lesions into long DNA and nucleosomes. CGRID uses paired Cas9 nickases to generate two adjacent nicks, followed by gap formation and replacement with a synthetic lesion-containing oligonucleotide. This design enables easy and efficient insertion of diverse DNA lesions at user-defined positions without pre-engineered nicking sites or substrate redesign. Applied to long λ-DNA, CGRID supports efficient installation of single lesions and internal double-strand breaks at predefined positions, which are challenging to obtain using existing methods. These molecules enable high-throughput single-molecule analysis of diverse repair pathways including base excision repair (BER), nonhomologous end joining, and microhomology-mediated end joining without hydrodynamic force. CGRID further allows efficient site-specific lesion incorporation into nucleosomal DNA, enabling systematic repositioning of damage within a nucleosome. This shows that nucleosome context and lesion orientation strongly influence APE1 incision efficiency. Together, CGRID establishes a broadly applicable platform for programmable DNA damage engineering, bridging biochemical and single-molecule methodologies, and facilitates mechanistic studies of chromatin-dependent genome maintenance.

## Graphical Abstract

**Graphical Abstract:**
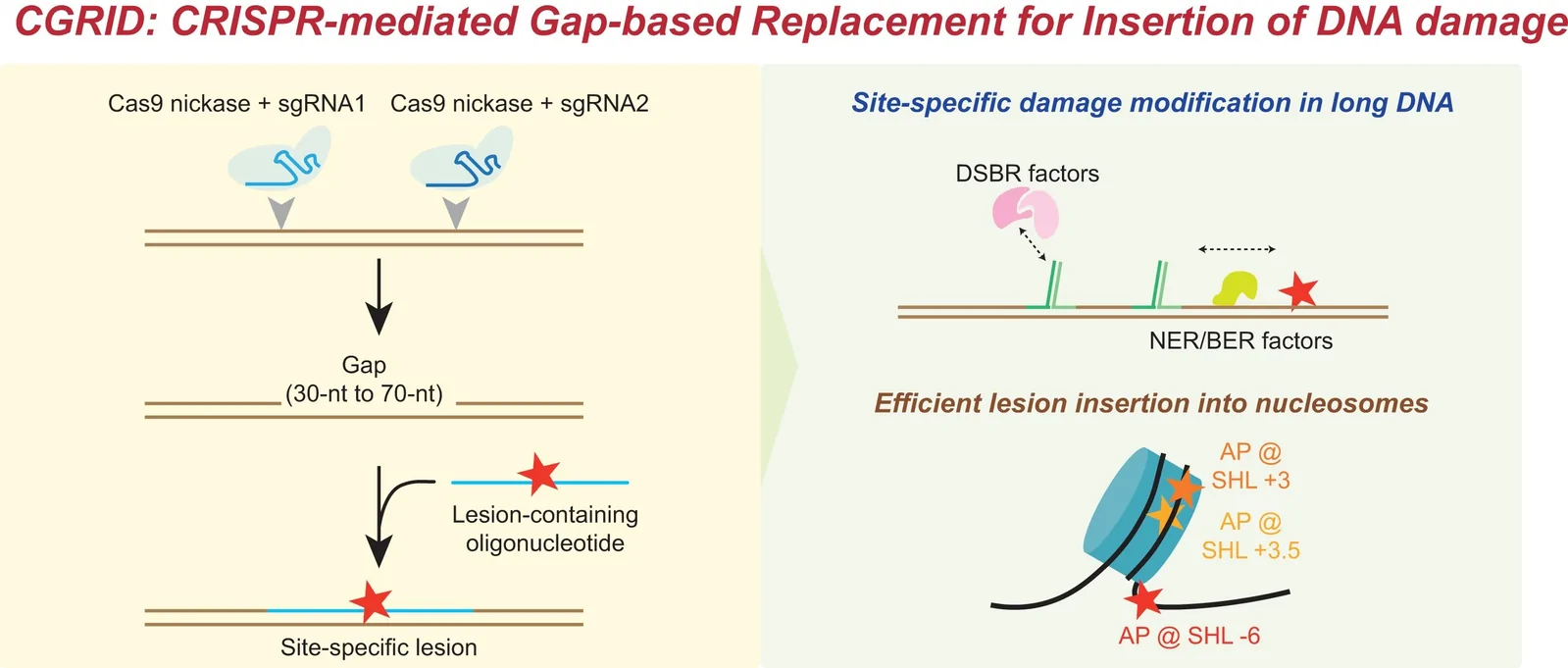

## Introduction

DNA damage arises continuously from endogenous metabolism, environmental stress, and genotoxic agents, and its accumulation threatens genome stability, accelerates aging, and contributes to human disease including cancer [1, 2]. Cells have therefore evolved multiple repair pathways that recognize and process chemically and structurally diverse lesions embedded in different DNA contexts [2, 3]. Mechanistic dissection of these pathways requires DNA substrates that contain defined lesions of known identity, copy number, and position.

This need is particularly acute in single-molecule and biochemical studies. Flow-stretched single-molecule platforms, including DNA curtains, have provided powerful insights into how repair proteins search for, recognize, and process damaged DNA [4–9]. These approaches commonly use lambda phage DNA (λ-DNA) because of its long length (48 502 bp) and experimental tractability. However, precise lesion engineering in long DNA remains a major technical limitation. Current strategies often rely on specially engineered λ-DNA substrates containing predefined nicking sites, which restrict lesion placement to fixed positions and, in some designs, introduce repeated lesions rather than a single defined damage site [10–13]. As a result, it remains challenging to install diverse DNA lesions flexibly and efficiently at arbitrary internal positions in long DNA molecules.

This limitation is especially problematic for studies of DNA double-strand breaks (DSBs), which are the most serious DNA damage, and precise engineering of such substrates remains challenging [14]. In many flow-based assays, one end of λ-DNA is tethered, whereas the other end remains free, allowing the free end to mimic a DSB [15–17]. Although useful, this configuration requires continuous hydrodynamic flow to maintain DNA extension, potentially influencing protein behavior on DNA. Double tethering can eliminate the hydrodynamic force, but it also removes access to a free DNA end and therefore complicates analysis of DSB repair. To circumvent this issue, bridged DNA containing internal DSBs was developed based on click chemistry, but its yield and generality have not been sufficient for high-throughput single-molecule imaging [18]. Recently, multiple DSBs were introduced into a single DNA molecule held by dual-bead optical traps, named as OT-Curtains [19]. However, this approach needed complicated plasmid cloning, fixed the positions of DSB sites, and permitted only a single, identical DSB configuration. A versatile strategy for introducing structurally defined DSBs at arbitrary internal positions in long DNA would therefore substantially expand the experimental scope of force-free single-molecule studies.

The same challenge extends beyond DSBs. A broad range of DNA lesions, including ultraviolet (UV) photoproducts, mismatches, oxidative lesions, and apurinic/apyrimidinic (AP) sites, have been investigated using biochemical and single-molecule approaches [13, 20, 21]. In most cases, lesion-containing substrates are generated by chemical synthesis of short oligonucleotides. While this provides precise control over lesion identity, the approach becomes inefficient, costly, or impractical as substrate length increases. Alternatively, long DNA can be damaged by physical or chemical treatment, such as UV irradiation, but such methods offer limited control over lesion type, number, and position [22]. Thus, a general method for installing defined lesions into long DNA substrates remains lacking.

An additional barrier is posed by chromatin. In eukaryotic cells, DNA damage occurs primarily in nucleosomal DNA, where histone association can strongly influence lesion accessibility and repair. Studying repair in a chromatin context therefore requires methods that can position defined lesions within nucleosomes or chromatin fibers. Existing approaches, including ligation-based assembly of lesion-containing Widom 601 substrates, are useful but often labor-intensive, position-restricted, and low yielding [23–27]. Changing lesion position typically requires synthesis of a new DNA construct, and extension to longer chromatin templates is even more challenging. Moreover, inserting a lesion into chromatin consisting of multiple nucleosomes is more difficult than for a nucleosome because the synthesis of longer oligomers is expensive or inefficient. Plug-and-play strategies have partially addressed this problem, but they generally require prior modification of the nucleosome-positioning sequence, which may affect nucleosome assembly or stability [28, 29].

Here, we present CGRID (CRISPR-mediated Gap-based Replacement for Insertion of DNA damage), a programmable strategy for installing defined DNA lesions into long DNA molecules and nucleosomes. CGRID uses paired Cas9 nickases (nCas9s) carrying the D10A mutation to generate two adjacent single-strand nicks at a user-defined position, followed by local gap formation and replacement with a synthetic lesion-containing oligonucleotide (Fig. 1A). Because targeting is determined by sgRNA design rather than by pre-engineered nicking sites, CGRID enables flexible and efficient insertion of diverse DNA lesions into substrates ranging from short DNA fragments to λ-DNA and nucleosomal DNA. Using this approach, we generate single AP-site substrates, internal DSBs, and multiple DSBs in long DNA for high-throughput single-molecule analysis, and we introduce lesions into nucleosomal DNA to examine how nucleosome position and orientation influence repair enzyme activity. CGRID therefore provides a general framework for programmable DNA damage engineering across biochemical and single-molecule platforms.

**Figure 1.**
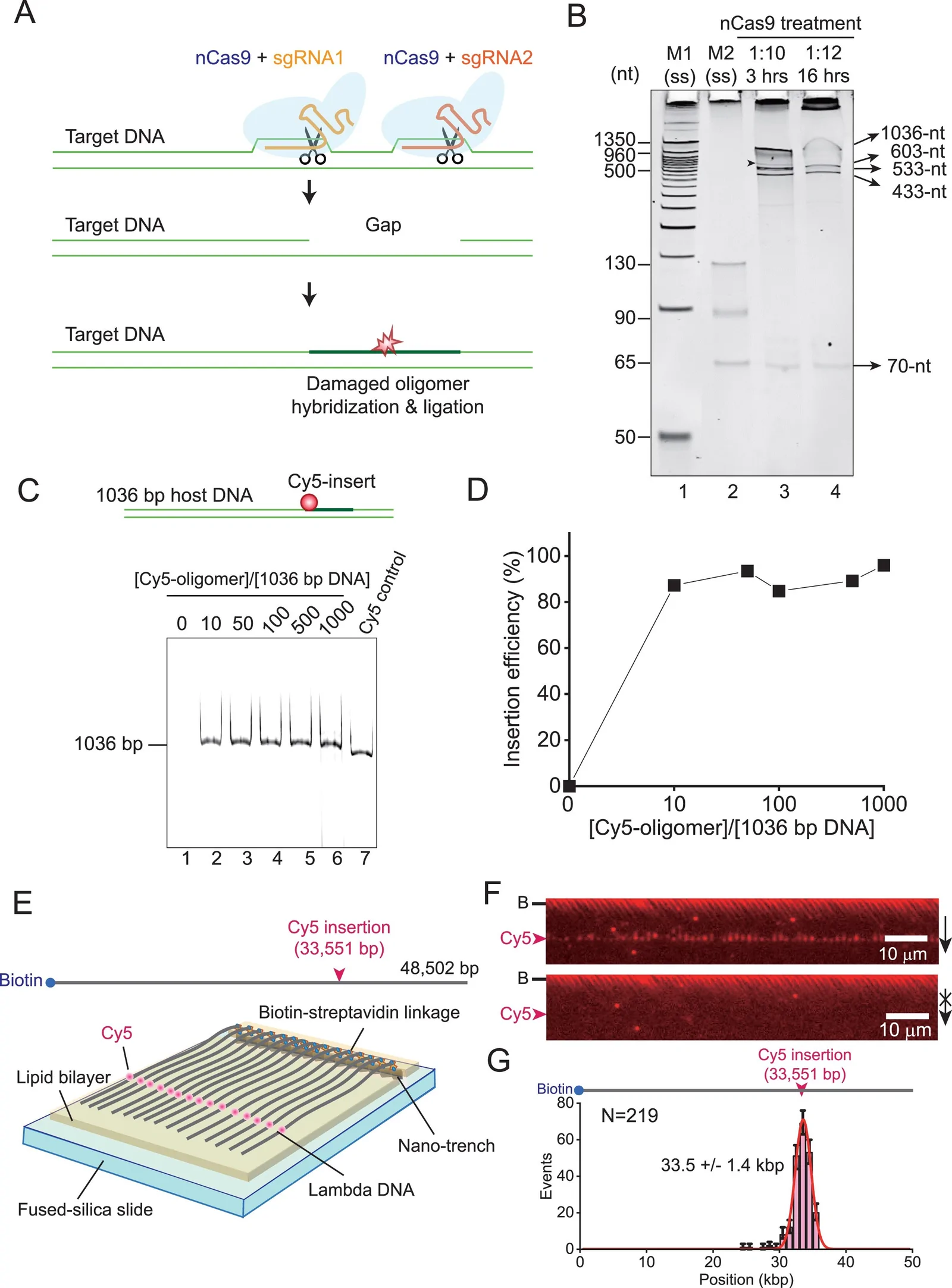
Proof of principle for damage insertion into DNA using nCas9. (**A**) Strategy for DNA damage insertion in the middle of DNA using two nCas9–sgRNA complexes. (**B**) Gap generation by treating two nCas9–sgRNA complexes with 1036-bp DNA. M1 and M2 represent single-stranded DNA ladders. Arrowhead indicates a DNA fragment (603-nt) cleaved by one nCas9–sgRNA complex. (**C**) Cy5-labeled oligomer insertion into nCas9-treated 1036-bp DNA. (Top) Schematic of Cy5 insertion into 1036-bp DNA. (Bottom) Gel image for Cy5-inserted 1036-bp DNA for different concentrations of Cy5-oligomers. Cy5 control in lane 7 indicates 10 nM PCR product of 1036-bp DNA with Cy5 at the 5′ end. (**D**) Insertion efficiency of Cy5 oligomers depending on the molar ratio of Cy5 oligomers to 1036-bp DNA. The insertion efficiency is estimated by comparing the fluorescence intensity with 10 nM Cy5 control. (**E**) Schematic of single-tethered DNA curtains, which are formed using λ-DNA containing internal Cy5 at 33 551 bp. (**F**) DNA curtain images showing internal Cy5 in the presence (top) and absence (bottom) of buffer flow. “B” at the left represents the barrier position, and Cy5 position is marked by an arrowhead. The arrow at the right indicates the orientation of buffer flow. (**G**) Distribution of Cy5 fluorescence puncta, which is fitted with a single Gaussian function. The peak center is 33.5 ± 1.4 kb. *N* represents the total number of analyzed molecules.

## Materials and methods

Details of materials and methods are described in Supplementary Information.

### DNA preparation

All oligomers used in this paper are listed in Supplementary Table S1.

To test the efficiency of DNA damage insertion, a 1036-bp DNA fragment was generated via PCR amplification using λ-DNA (New England Biolabs, N3011S) as a template with primers (1036F and 1036R) (Supplementary Table S1). To insert a lesion into Widom 601 DNA, a plasmid containing Widom 601 sequence was provided by Professor Hajin Kim at Ulsan National Institute of Science and Technology, South Korea. A 200-bp DNA fragment having Widom 601 sequence was generated via PCR using the plasmid as a template with primers (CY5-200 bp-F and 200 bp-R) (Supplementary Table S1). A 149-bp DNA fragment having Widom 601 sequence was generated via PCR using the plasmid as a template with primers [CY5-149 bp-F (or 149 bp-F) and CY5-149 bp-R (or 149 bp-R)] (Supplementary Table S1). All PCR products were purified using the Expin™ PCR SV Kit (Gene All, 103-102).

For DNA curtains, λ-DNA was ligated with 500-fold excess of oligomers containing biotin or digoxigenin (Lambda biotin-L/Lambda dig-R: BLDR or Lambda biotin-R/Lambda dig-L: BRDL) as described in the previous studies [30, 31] (Supplementary Table S1).

### Protein preparation

#### Purification of Cas9 D10A nickase

Purification of Cas9 D10A nickase (nCas9) followed the previous protocol with slight modifications [32–34]. The detailed protocol is described in Supplementary Information. Briefly, nCas9 with 6× His and 1× HA tags at the amino terminus was overexpressed in *E. coli* BL21 (DE3) at 25°C for 4 h. Cells were lysed in nCas9 lysis buffer [50 mM NaH_2_PO_4_ (8.0), 300 mM NaCl, 10 mM imidazole, 10% glycerol, 1 mM DTT, HALT Protease Inhibitor Cocktail (Thermo Fisher Scientific, 78438), and 1 mM PMSF (phenylmethanesulfonyl fluoride)] by two-step sonication (Q700, Qsonica). Sonication was first applied at 30% amplitude with total on time of 1 min (10 s pulse on and 15 s pulse off), and then the total on time was changed to 10 min (1 s pulse on and 10 s pulse off). Cell lysates were clarified, and then nCas9 was successively purified by 5 ml of HisPur™ Ni-NTA Resin (Thermo Fisher Scientific, 88222) and a HiTrap™ Heparin HP 5 ml column (Cytiva, 17040701). Finally, the protein was dialyzed against nCas9 storage buffer [20 mM HEPES–NaOH (7.5), 150 mM KCl, 10% glycerol, and 1 mM DTT] at 4°C using Spectra/Por™ 4 dialysis tubing (12–14 kDa MWCO, Spectra/Por, 132706) (Supplementary Fig. S1A). The final sample was snap-frozen in liquid nitrogen and then stored at −80°C until use.

#### APE1 purification and activity test

A 6× His tag and a 3× HA tag were fused to the amino- and carboxyl-terminus of wild-type APE1 and a catalytically inactive mutant APE1_D210N, respectively. Both wild-type and D210N mutant were purified using the same protocol. APE1 proteins were overexpressed in *E. coli* BL21 (DE3) strain at 16°C overnight. Cells were harvested and lysed by sonication, and the cell lysates were clarified by ultracentrifugation at 100 000 *g* at 4°C for 1 h. The clarified supernatant was loaded onto a 1 ml HisTrap HP column (Cytiva, 17524701) pre-equilibrated with lysis buffer [20 mM Tris–HCl (7.5), 250 mM NaCl, 1× HALT protease (Thermo Fisher Scientific, 78438), 1 mM PMSF, and a cOmplete tablet (Roche, 11836170001)]. The column was washed with 25 ml of wash buffer [20 mM Tris–HCl (7.5) and 1 M NaCl]. The protein was eluted using a linear imidazole gradient from 0 to 500 mM in elution buffer [20 mM Tris–HCl (7.5) and 250 mM NaCl]. The 6× His tag was eliminated by TEV protease cleavage during dialysis against Storage buffer [20 mM Tris–HCl (7.5), 250 mM NaCl, 10 mM βME (β-mercaptoethanol), and 20% glycerol]. Cleaved 6× His tag and TEV protease were removed through Ni-NTA resin (Thermo Fisher Scientific, 88222). The final purified APE1 protein was stored in Storage buffer. Enzyme activity was assessed by cleavage of AP site-containing DNA substrates (Supplementary Fig. S2A and B).

#### Polθ purification and activity test

A truncated version of the human Polθ protein encompassing residues 1792–2590 (Polθ_QM1) tagged with a 10× His-GST tag at the amino terminus was purified following a previously described protocol [35] (Supplementary Fig. S3A). Details of purification are described in Supplementary Information. Briefly, cells grown at 20°C for 60 h were resuspended in five volumes of equilibration buffer [50 mM HEPES–KOH (8.0), 300 mM NaCl, 5 mM CaCl₂, 20 mM imidazole, 10% glycerol, 1.5% NP-40, 5 mM βME, 100 µg/ml lysozyme, and EDTA-free protease inhibitor mixture (Roche, 04693132001)]. The resuspended cells were sonicated and then clarified. The protein was purified by 5 ml of His-Tag Purification Resin (Roche, 08778850001) and 1 ml of Glutathione Sepharose 4B. After buffer change to PreScission cleavage buffer [50 mM Tris–HCl (6.8), 150 mM NaCl, 1 mM DTT, 1 mM EDTA, and 0.01% NP-40] in an Econo-Pac 10DG column (Bio-rad, 7321010), the His-GST tag was then cleaved by adding 20 µl of PreScission Protease and incubating at 4°C for 4 h. The cleaved His-GST tag was removed by 500 µl of His-Tag Purification Resin followed by 500 µl of Glutathione Sepharose 4B. The protein was then further purified by a Mono S 5/50 GL column (Cytiva, 17516801). After buffer exchange, the sample was purified through a HiTrap Heparin HP column (Cytiva, 17040701). The final fractions were buffer-exchanged into Polθ reaction buffer [37.5 mM Tris–HCl (8.0), 40 mM NaCl, 6.25% glycerol, 2.5 mM DTT, and 0.01% Triton X-100] using an Econo-Pac 10DG column, concentrated with an Amicon Ultra-0.5 ml 50K centrifugal filter unit (Millipore, UFC5050), and stored at −80°C. The enzymatic activity of purified Polθ_QM1 was tested using Insert_pol θ_3 and Insert_pol θ_4, which contain microhomology (Supplementary Fig. S3B and C).

#### Human Ku70/80 purification and activity test

Human Ku70/80 was purified by the previous protocol with slight modifications [36]. The detailed protocol is described in Supplementary Information. Briefly, Ku70 without any tag and Ku80 with 6× His and 3× FLAG tag at the amino terminus were overexpressed in *E. coli* Rosetta2 (DE3) pLysS at 16°C. Cells were lysed in 70 ml Ku lysis buffer [50 mM Tris–HCl (8.0), 1 M NaCl, 5% glycerol, 0.4 M ammonium acetate, 10 mM imidazole, 2 mM βME, 1 mM PMSF, 1× cOmplete EDTA-free Protease Inhibitor Cocktail (Roche, 11873580001), and 0.2 mg/ml lysozyme] by 30% amplitude sonication (Q700, Qsonica) with total on time of 5 min (5 s pulse on and 1 min pulse off). The clarified lysates were successively purified by 10 ml of TALON Superflow Metal Affinity Resin (Takara, 635670), Superdex 200 Increase 10/300 GL column (Cytiva, 28990944), and Mono Q® 5/50 GL column (Cytiva, 17516601). Finally, the proteins were dialyzed against Ku Storage buffer [50 mM Tris–HCl (8.0), 50 mM NaCl, 20% glycerol, 0.5 mM EDTA, 1 mM PMSF, and 1 mM DTT] at 4°C using Spectra/Por™ 4 dialysis tubing (12–14 kDa MWCO, Spectra/Por, 132706). The activity of purified Ku70/80 complexes was tested by binding to DNA ends (Supplementary Fig. S3D and E).

### Histone purification and *in vitro* nucleosome reconstitution

*In vitro* nucleosome reconstitution was conducted using *Xenopus laevis* canonical histones (H2A, H2B, H3, and H4). Detailed protocols for the purification of the histones, octamer assembly, and nucleosome reconstitution are described in Supplementary Information [37, 38]. The reconstituted nucleosomes were tested by gel shift assay and micrococcal nuclease (MNase) assay. The details are described in Supplementary Information.

### CRISPR–Cas9 nickase treatment for CGRID

#### *In vitro* transcription for single-guide RNA

The template DNA for *in vitro* transcription (IVT) was obtained by polymerase chain reaction (PCR) using the spCas9 Universal primer (IVT_Universal) and a primer containing target sequence (IVT_right_1, IVT_right_2, IVT_middle_1, IVT_middle_2, IVT_left_1, IVT_left_2, IVT_Widom_+3/+3.5_1, IVT_Widom_+3/+3.5_2, or IVT_Widom_−6) (Supplementary Fig. S1B and C and Supplementary Table S1). PCR products were purified using the Expin™ PCR SV Kit (Gene All, 103-102). IVT reaction was performed using the HiScribe T7 Quick High Yield RNA Synthesis Kit (New England Biolabs, E2050S) with each template DNA. The reaction mixture, consisting of 20 µl of NTP buffer, 4 µl of T7 RNA polymerase, 2 μg of IVT template DNA, and diethyl pyrocarbonate (DEPC)-treated deionized water (DI) in a final volume of 60 µl, was incubated at 37°C for 4 h. Subsequently, 4 µl of DNase I (New England Biolabs, M0303S) and 36 µl of DEPC-treated DI were added to the reaction mixture, which was incubated at 37°C for 30 min. The synthesized sgRNA was purified using the Monarch® RNA Cleanup Kit (New England Biolabs, T2040L). The synthesis of sgRNA was confirmed by 8 M urea denaturing Polyacrylamide gel electrophoresis (PAGE) (Supplementary Fig. S1D). The names of sgRNAs generated using each primer are listed in Supplementary Table S2.

#### CRISPR–Cas9 nickase reaction for 1036 bp PCR product from λ-DNA

Before nicking reaction for λ-DNA, we treated purified nCas9 with a 1 kb PCR product derived from λ-DNA (Fig. 1B). A total of 360 nM of nCas9 and 360 nM of sgRNA_right_1 (or sgRNA_right_2) were mixed and incubated at 23°C for 15 min. For nicking reaction, the two RNA–protein (RNP) complexes and 30 nM of the 1 kb PCR product were mixed and incubated in 1× NEBuffer r3.1 (New England Biolabs, B7203S) at 37°C for 16 h. The reaction was stopped by adding 0.3 mg/ml Proteinase K and 0.6 mg/ml RNase A (Merck, R4875) to the mixture, followed by incubation at 37°C for 30 min. Proteinase K was then heat-inactivated at 80°C for 20 min.

#### Gap generation in λ-DNA by nCas9

Nine nanomolar BRDL or BLDR λ-DNA prepared as described above was treated with 108 nM nCas9 together with 108 nM each of sgRNA_right_1 and sgRNA_right_2 to generate a 70-nt gap at 33.5 kb in BRDL λ-DNA or at 14.9 kb in BLDR λ-DNA. Separately, 9 nM BRDL λ-DNA was treated with 108 nM nCas9 together with 108 nM of sgRNA_middle_1 and sgRNA_middle_2 to generate a 66-nt gap at 21.6 kb.

For triple gaps, 108 nM nCas9 was preincubated with 108 nM of each of six sgRNAs (sgRNA_right_1, sgRNA_right_2, sgRNA_middle_1, sgRNA_middle_2, sgRNA_left_1, and sgRNA_left_2). Nine nanomolar BRDL (or BLDR) λ-DNA was treated with all the six RNPs together. The mixture was incubated in 1× NEBuffer r3.1 (New England Biolabs, B7203S) at 37°C for 16 h. The reaction was stopped by 30 min incubation with 0.6 mg/ml RNase A (Merck, R4875) at 37°C. Subsequently, to remove RNase A and nCas9, 0.3 mg/ml proteinase K was added and incubated at 37°C for 30 min. Proteinase K was finally heat-inactivated at 80°C for 20 min.

#### Gap generation in Widom 601 DNA by nCas9

To generate a 30-nt gap within 200 bp Widom 601 DNA or at the superhelical location (SHL) +3 or SHL +3.5 position of 149 bp Widom 601 DNA, nCas9 was sequentially treated. For the first nick, 600 nM of nCas9 and 600 nM of sgRNA_Widom_+3/+3.5_1 were incubated at 23°C for 15 min. The first RNP complex was mixed with 50 nM Widom 601 DNA (200 or 149 bp) in 1× NEBuffer r3.1 (New England Biolabs, B7203S). In this reaction, 30 nM pUC19 plasmid was also added as a competitor to reduce off-target effects. The mixture was incubated at 37°C for 16 h. The reaction was stopped by heat inactivation at 65°C for 20 min. For the second nick, 300 nM of nCas9 and 300 nM of sgRNA_Widom_+3/+3.5_2 were incubated at 23°C for 15 min. This second RNP complex was added to the 25 nM of Widom 601 DNA (200 or 149 bp) that was treated with the first RNP. At this point, the sample volume was doubled. The mixture was incubated at 37°C for 16 h.

For a 32-nt gap at SHL −6 position of 149 bp Widom DNA, 600 nM of nCas9 and 600 nM of sgRNA_Widom_−6 were preincubated at 23°C for 15 min. Then, 50 nM of 149 bp Widom DNA, and 30 nM pUC19 plasmid were added in 1× NEBuffer r3.1 buffer (New England Biolabs, B7203S). The mixture was incubated at 37°C for 16 h.

All reactions were stopped by 30-min incubation with 0.6 mg/ml RNase A (Merck, R4875) at 37°C. Subsequently, to remove RNase A and nCas9, 0.3 mg/ml proteinase K was added and incubated at 37°C for 30 min. Proteinase K was finally heat-inactivated at 80°C for 20 min.

### Oligomer insertion into a gap formed by Cas9 nickases for CGRID

#### Cy5-oligomer insertion into a 70-nt gap in the 1036 bp PCR product

To anneal Cy5-labeled oligonucleotides into the 70-nt gap of the 1036 bp PCR product, 10 nM of the gapped DNA substrate was mixed with Insert_right_Cy5 oligonucleotides at 10-, 50-, 100-, 500-, or 1000-fold excess. The mixture was heated to 95°C for 10 min and then slowly cooled down to 23°C.

#### Insertion of double-strand break construct into a 70-nt gap generated by Cas9 nickases

For the annealing of CRISPR-treated BLDR λ-DNA (or BRDL λ-DNA) with oligonucleotides, 4 nM of CRISPR-treated BLDR λ-DNA was mixed with a 500-fold excess of Insert_DSB_2 and Insert_DSB_3 in T4 ligase buffer (Enzynomics, B304). A 500-fold excess of oligomers was sufficient for efficient DSB insertion without any purification of λ-DNA following CRISPR treatment. The mixture was heated to 80°C for 10 min and then allowed to slowly cool down to 23°C for annealing. For nick sealing, the annealed DNA was treated with T4 DNA ligase (Enzynomics, M001) and incubated at 23°C for 16 h. After incubation, the enzyme was heat-inactivated at 65°C for 20 min. The λ-DNA was then purified using an S-400 spin column (GE Healthcare, Illustra MicroSpin S-400).

Before being inserted into λ-DNA, Insert_DSB_1 (or Insert_DSB_1_internal Cy5) and Insert_DSB_4 were hybridized by heating a mixture of the two oligomers at 95°C for 10 min followed by slowly cooling down to 23°C. Subsequently, a 1000-fold excess of the prehybridized Insert_DSB_1 (or Insert_DSB_1_internal Cy5)/Insert_DSB_4 duplex oligonucleotide was added to the purified λ-DNA in T4 ligase buffer. The mixture was heated to 70°C for 10 min and then slowly cooled down to 23°C for annealing. For the final nick sealing, the DNA was treated with T4 DNA ligase (Enzynomics, M001) and incubated at 23°C for 16 h, followed by heat inactivation at 65°C for 20 min. λ-DNA containing an internal DSB was then purified using an S-400 spin column (GE Healthcare, Illustra MicroSpin S-400).

#### Insertion of a single AP-site or Cy5-labeled oligonucleotide into λ-DNA

For single AP-site insertion into λ-DNA, 4 nM of λ-DNA containing a 70-nt gap was mixed with a 500-fold excess of Insert_AP. For single Cy5 fluorescence insertion into λ-DNA, 4 nM of λ-DNA containing a 70-nt gap at 33.5 kb was mixed with a 500-fold excess of Insert_right_Cy5. In addition, 4 nM of λ-DNA containing a 66-nt gap at 21.6 kbp was mixed with a 500-fold excess of Insert_middle_Cy5. The mixture was heated up to 80°C for 20 min and then slowly cooled down to 23°C to allow annealing. The gap was subsequently sealed with T4 DNA ligase (Enzynomics, M001) in 1× T4 ligase buffer (Enzynomics, B304) by incubation at 23°C for 16 h, followed by heat-inactivation at 65°C for 20 min. λ-DNA containing the AP site was then purified using an S-400 spin column (GE Healthcare, Illustra MicroSpin S-400).

#### Insertion of double-strand break containing microhomology into λ-DNA

For an internal DSB containing an ssDNA overhang with microhomology, 4 nM of λ-DNA containing a 70-nt gap was mixed with a 500-fold excess of Insert_pol θ_1, and Insert_pol θ_2. The mixture was heated up to 80°C for 20 min and then slowly cooled down to 23°C for annealing. Then, T4 DNA ligase (Enzynomics, M001) sealed the gap in 1× T4 ligase buffer (Enzynomics, B304) by incubating at 23°C for 16 h and then was heat-inactivated at 65°C for 20 min. The λ-DNA containing an internal DSB with microhomology was purified using an S-400 spin column (GE Healthcare, Illustra MicroSpin S-400).

#### Insertion of triple double-strand breaks into λ-DNA

To insert triple DSBs into λ-DNA, 4 nM λ-DNA containing triple gaps was mixed with 500-fold excess of Insert_TD_1A, Insert_TD_1B, Insert_TD_2A, Insert_TD_2B, Insert_TD_3A, and Insert_TD_3B. Then, the mixture was heated up to 80°C for 20 min and allowed to slowly cool down to 23°C for annealing. Nicks were sealed by T4 DNA ligase (Enzynomics, M001) in 1× T4 ligase buffer (Enzynomics, B304) at 23°C for 16 h. T4 DNA ligase was heat-inactivated at 65°C for 20 min. Excessive oligomers were removed by an S-400 spin column (GE Healthcare, Illustra MicroSpin S-400).

#### Insertion of modified oligomers into gapped Widom 601 DNA

For insertion of modified oligomers into gapped Widom 601 DNA, 50 nM of nCas9-treated 200 bp or 149 bp Widom 601 DNA was first mixed with a five-fold excess of the corresponding complementary oligomer (Complementary_SHL +3/+3.5 or Complementary_SHL−6) to expose the gap more efficiently by preventing heat-denatured single-stranded DNA from re-annealing with the gap. The mixture was heated to 95°C for 20 min and then slowly cooled to 65°C.

For the 30-nt gap in 200 bp Widom 601 DNA, a 200-fold excess of an oligomer containing fluorescein-dT or AP (Insert_Widom_fluorescein, Insert_Widom_+3AP, or Insert_Widom_+3.5AP) was annealed. The mixture was heated up to 65°C for 20 min, then slowly cooled down to 23°C.

For AP insertion into 149 bp Widom 601 DNA, a 200-fold excess of an AP-containing oligomer (Insert_Widom_+3AP, Insert_Widom_+3.5AP, or Insert_Widom_−6AP) was added and incubated at 65°C for 20 min, followed by slow cooling to 23°C.

For sealing nicks, T4 DNA ligase was incubated at 23°C for 16 h and then heat-inactivated at 65°C for 20 min. The pUC19 plasmid competitors were removed by 6% acrylamide and 0.5× TBE gel using Mini Prep Cell (Bio-rad, 1702928) and then purified Widom 601 DNA was concentrated using an Expin™ PCR SV Kit (Gene All, 103-102).

### DNA curtain assay

All DNA curtain experiments were performed at 23°C. DNA curtains were conducted by following previous studies [5, 9, 10, 39, 40, 41]. All details for DNA curtain assays are described in Supplementary Information.

### Biochemical assays

#### Fluorescein insertion efficiency

To assess the fluorescein insertion efficiency in the 200 bp Widom 601 DNA, 10 nM Widom 601 DNA containing fluorescein was incubated with 0.1 μM or 1 μM antifluorescein antibody (Jackson Immunoresearch, 200-002-037) in 10 mM Tris–HCl [8.0] and 0.1 mM EDTA at 23°C for 30 min. The reactions were then analyzed using a 6% native polyacrylamide gel in 1× TBE at 4°C, which was imaged using a Typhoon RGB scanner (Cytiva).

To examine the insertion of fluorescein into a nucleosome, 10 nM fluorescein-containing nucleosomes were incubated with 10, 20, and 30 nM antifluorescein antibody (Jackson Immunoresearch, 200-002-037) in 10 mM Tris–HCl [8.0] and 0.1 mM EDTA at 23°C for 30 min. The gel shift assay was analyzed by 5% native PAGE in 0.5× TBE at 4°C for 60 min. The gel was imaged using a Typhoon RGB scanner (Cytiva).

#### APE1 cleavage test

To examine the cleavage efficiency of APE1 at AP sites in naked DNA and nucleosomes depending on AP-site position, 10 nM AP-containing DNA or nucleosome was incubated with APE1 at 0.1, 0.3, 1, 3, and 10 nM in reaction buffer containing 20 mM Tris–HCl (7.5), 50 mM NaCl, 1 mM MgCl_2_, 1 mM DTT, and 0.1 mg/ml BSA at 23°C for 10 min. Reactions were quenched immediately by adding an equal volume of stop solution containing 95% formamide and 10 mM EDTA. The samples were then boiled at 95°C for 10 min to denature all DNA oligomers. The reaction products were analyzed on 10% or 12% denaturing PAGE gels containing 8 M urea in 0.5× TBE buffer at 55°C for 20–30 min. Gels were imaged using a Typhoon RGB scanner (Cytiva).

#### SMARCA4 remodeling assay

To test nucleosome remodeling by recombinant SMARCA4 (EpiCypher, 15-1014), 200 bp AP-containing nucleosomes were used because SMARCA4 exhibited no detectable remodeling activity toward 149 bp nucleosomes lacking flanking DNA. A total of 10 nM of 200 bp AP-containing nucleosomes were incubated with 1, 3, and 5 nM SMARCA4 in SMARCA4 reaction buffer containing 20 mM Tris–HCl (7.5), 50 mM NaCl, 1 mM MgCl_2_, 1 mM DTT, 0.1 mg/ml BSA, and 1 mM ATP at 23°C for 30 min. Reactions were stopped by adding 10 mM EDTA. The reactants were run on 7% native PAGE in 0.5× TBE at 4°C for 90 min. The gel was analyzed using a Typhoon RGB scanner (Cytiva).

To examine whether APE1 cleavage efficiency of AP sites in nucleosomes is altered by SMARCA4, 10 nM of 200 bp AP-containing nucleosomes were reacted with 3 nM SMARCA4 in SMARCA4 reaction buffer at 23°C for 20 min. After the SMARCA4 reaction, 0.1, 0.3, 1, 3, or 10 nM of APE1 was incubated at 23°C for 10 min. The reactions were quenched by addition of an equal volume of stop solution containing 95% formamide and 10 mM EDTA, followed by heating at 95°C for 10 min. The reactants were run on 10% denaturing PAGE gels containing 8 M urea in 0.5× TBE buffer at 55°C for 30 min. Gels were analyzed using a Typhoon RGB scanner (Cytiva).

## Results

### CGRID enables efficient oligonucleotide replacement at user-defined positions in DNA

To establish a programmable strategy for DNA lesion installation, we designed a workflow in which two nCas9–sgRNA complexes generate adjacent single-strand nicks on the same strand of a target DNA molecule, thereby creating a short gap after heat denaturation of the intervening fragment. A complementary synthetic oligonucleotide carrying a desired modification is then annealed and ligated into the gap (Fig. 1A). We purified SpCas9 D10A nickase (nCas9) and generated sgRNAs by IVT (Supplementary Fig. S1).

As proof of principle, we tested this approach on a 1036-bp DNA substrate using a 70-nt Cy5-labeled oligonucleotide as a replacement fragment. Under optimized reaction conditions, paired nicking occurred efficiently, and subsequent oligonucleotide insertion proceeded with an efficiency of ∼90%, largely independent of the molar excess of the replacement oligonucleotide (Fig. 1B–D). The insertion efficiency was calculated from the ratio of the fluorescence intensity of Cy5-inserted DNA to that of the Cy5-labeled control DNA (lane 7 in Fig. 1C). We next applied the same strategy to λ-DNA and visualized the inserted Cy5 signal using single-tethered DNA curtains (Fig. 1E). The fluorescence signal localized at the designed position, confirming successful installation in a long DNA molecule (Fig. 1F and G). Re-targeting the system to a second site at 21.6 kb by changing the sgRNAs yielded efficient insertion at the expected position, indicating that the method is readily reprogrammable and is not restricted to a fixed lesion cassette (Supplementary Fig. S4). Together, these data establish that CGRID supports efficient and position-flexible installation of defined oligonucleotide inserts into long DNA.

### A single AP site installed by CGRID supports quantitative analysis of APE1 target search

To determine whether CGRID-generated substrates are suitable for site-specific insertion into long DNA for single-molecule studies, we introduced a single AP site into λ-DNA at position 33 555 bp and assembled double-tethered DNA curtains (Fig. 2A). Unlike previously used substrates containing multiple lesions at defined positions or lesions formed at random locations [10, 13, 42], CGRID enabled analysis of protein behavior on a single, site-specific lesion embedded in long DNA. We monitored lesion search by catalytically inactive APE1_D210N, which preserves lesion recognition while preventing incision (Supplementary Fig. S2).

**Figure 2.**
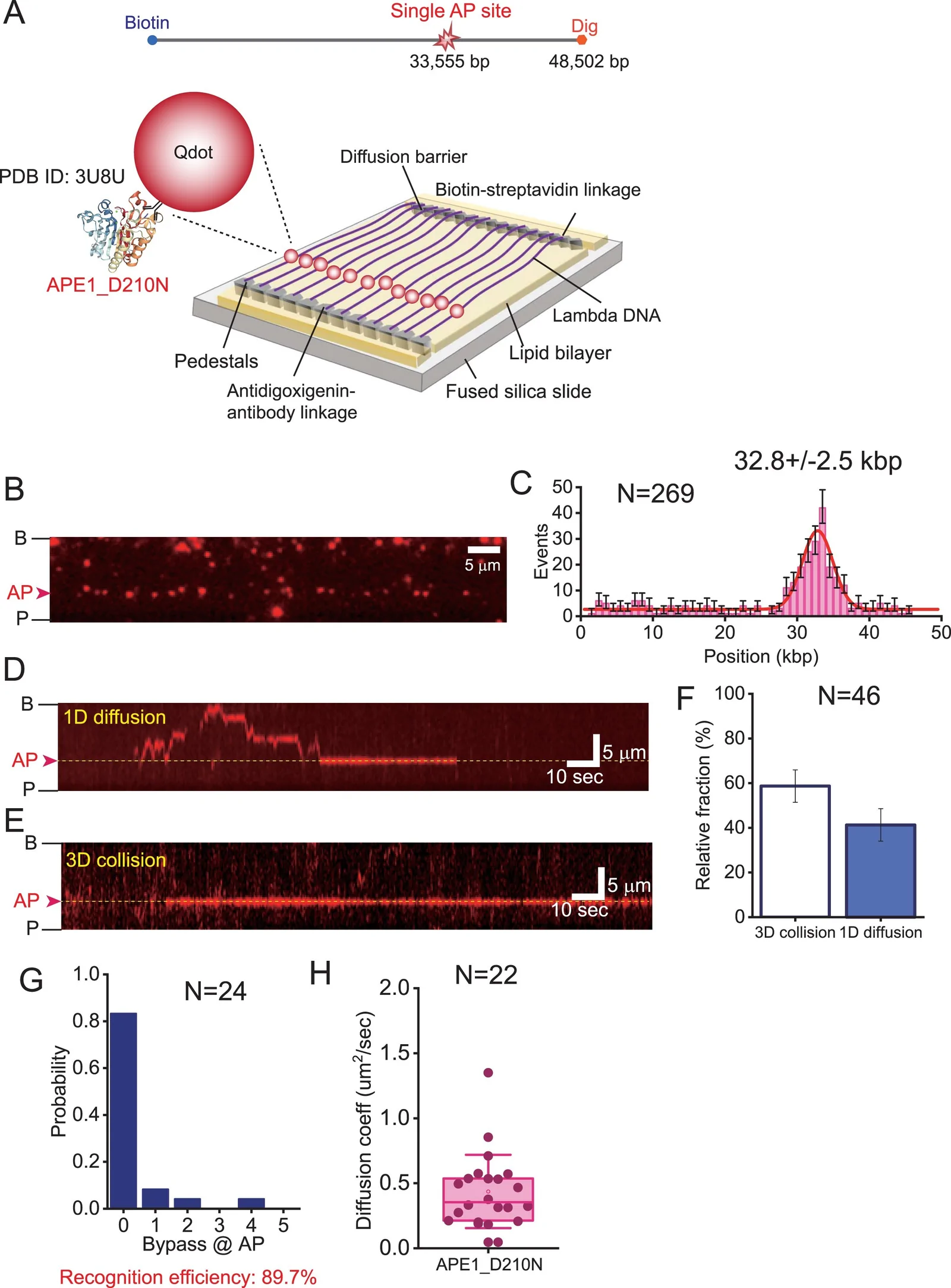
Insertion of a single DNA lesion into λ-DNA and its interaction with a repair protein. (**A**) Schematic of double-tethered DNA curtains built up with λ-DNA containing a single AP site at 33 555 bp of λ-DNA. APE1_D210N is conjugated with quantum dot (Qdot). The structure of APE1 is adopted from PDB (3U8U). (**B**) DNA curtain image showing preferential binding of APE1_D210N. (**C**) Binding distribution histogram of APE1_D210N. The histogram is fitted with a single Gaussian function with a peak at 32.8 ± 2.5 kb, which matches the AP insertion position. *N* represents the total number of analyzed molecules. (**D**) Representative kymograph showing one-dimensional (1D) diffusion of APE1_D210N to search for a single AP site. B and P at the left indicate the barrier and pedestal, respectively. Arrowhead and dashed line indicate the position of the single AP site. (**E**) Representative kymograph showing three-dimensional (3D) collision of APE1_D210N to search for a single AP site. B and P at the left indicate the barrier and pedestal, respectively. Arrowhead and dashed line indicate the position of the single AP site. (**F**) Relative population of 3D collision and 1D diffusion. Error bar is obtained from standard deviation of binomial distribution. N represents the total number of analyzed molecules. (**G**) Probability distribution of bypasses of APE1_D210N at the single AP site during 1D diffusion. *N* represents the total number of analyzed molecules. (**H**) Diffusion coefficients of APE1_D210N at 25 mM NaCl and 0.1 mM MgCl_2_. *N* represents the total number of analyzed molecules. Error bar is obtained from standard deviation.

Qdot-conjugated APE1_D210N accumulated specifically at the engineered AP site, producing a distinct localized binding peak in the positional distribution (Fig. 2B and C). Trajectory analysis revealed that APE1_D210N reached the lesion through both 1D diffusion and 3D collision (Fig. 2D and E). The apparent contribution of 3D collision (27 out of 46: 58.7% ± 7.3%) exceeded that of 1D diffusion (19 out of 46: 41.3% ± 7.3%), although short diffusive excursions below the spatial resolution of the assay were likely undercounted (Fig. 2F). These results are consistent with previous single-molecule studies demonstrating that APE1 searches for AP sites through a combination of 1D diffusion and 3D collision [43, 44]. However, the previous studies reported that 1D diffusion is the dominant search mode. This discrepancy may reflect differences in salt conditions. We then quantified lesion recognition during 1D diffusion by measuring bypass frequency. Most molecules recognized the AP site without bypass, yielding an estimated recognition efficiency of 89.7% (Fig. 2G). The diffusion coefficient of APE1_D210N was 0.44 ± 0.28 μm²/sec, which is consistent with the previous ones (Fig. 2H) [43, 44]. These results show that CGRID-generated single-lesion substrates support quantitative analysis of target search and lesion recognition with positional precision in long DNA.

### CGRID generates structurally defined internal DSBs in long DNA

We next attempted to incorporate a DSB substrate, the most severe DNA damage, into λ-DNA [14]. To do so, we hybridized two oligonucleotides to 70-nt gap generated by paired nicking. These oligonucleotides formed a Cy3-labeled primer-template junction containing a 30-nt single-stranded segment and a 20-bp duplex stem (Fig. 3A). In DNA curtain experiments, Cy3 fluorescence puncta localized to the designed insertion site at ∼15 kb, confirming successful formation of the internal DSB intermediate (Supplementary Fig. S5A and B). When the polarity of λ-DNA tethering was reversed, the fluorescence position shifted accordingly, further confirming correct internal placement of the DSB structure (Supplementary Fig. S5B and C). We then converted this intermediate into a blunt-ended internal DSB by annealing a Cy5-labeled complementary oligonucleotide to the single-stranded region (Fig. 3A). The Cy5 signal colocalized with the Cy3 signal and mapped to the designed target site, indicating successful formation of a blunt-ended internal break within λ-DNA (Fig. 3B and C).

**Figure 3.**
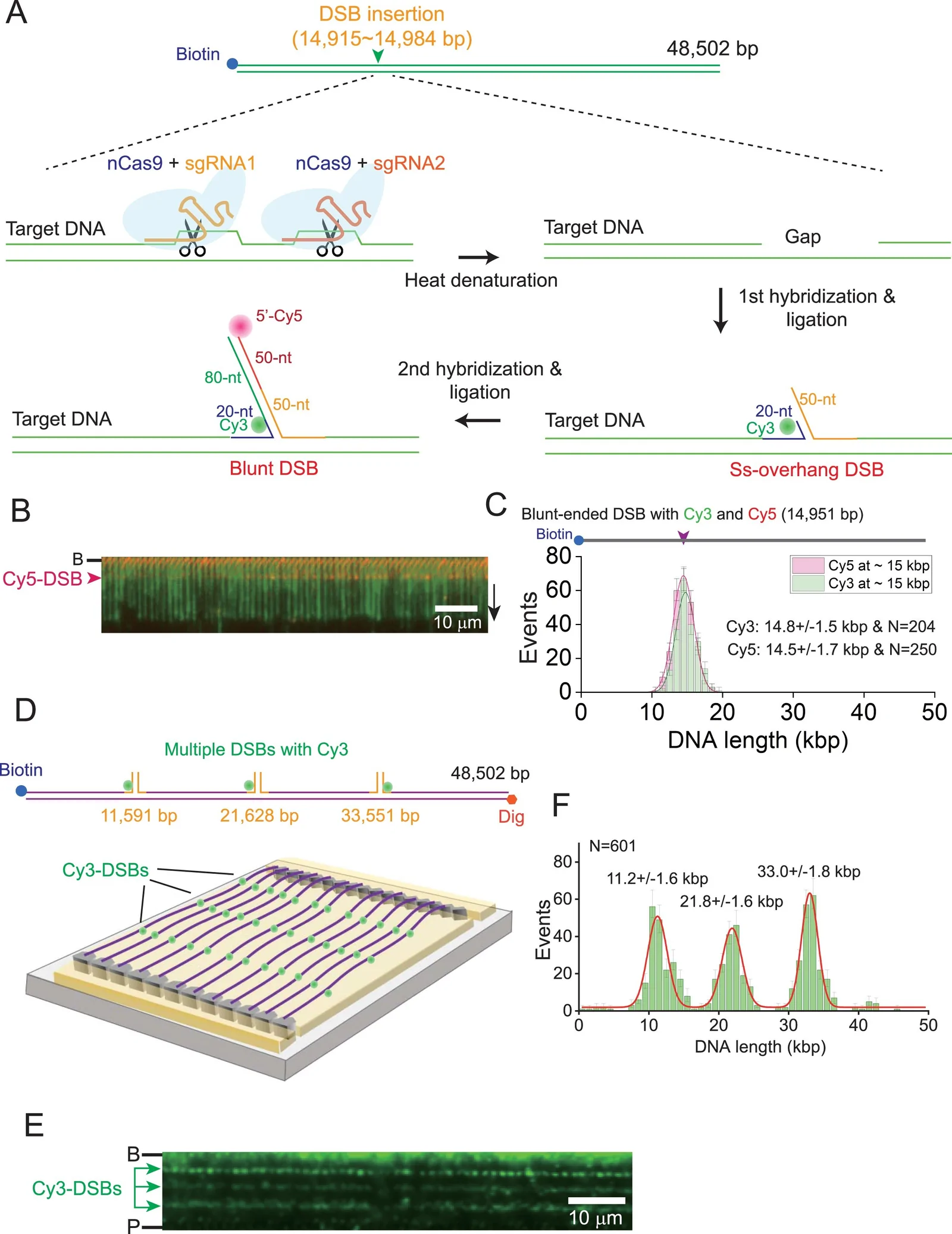
DNA DSB in the middle of λ-DNA. (**A**) (Top) Schematic of λ-DNA containing blunt-ended DSB. (Bottom) Strategy for the insertion of a blunt-ended DNA DSB into λ-DNA using nCas9 complexes. (**B**) DNA curtain image with λ-DNA containing Cy5-labeled DSB, the position of which is denoted by arrowhead. DNA is stained with YOYO-1. “B” at the left and the arrow at the right indicate the barrier position and flow direction, respectively. (**C**) Position distribution of Cy5 fluorescence puncta from Fig. 3B. There is one distinct peak at 14.5 ± 1.7 kb from fitting with a single Gaussian function, which overlaps well with Cy3 peak at 14.8 ± 1.5 kb (Supplementary Fig. S5B and C), indicating that the DSB is well inserted into λ-DNA. Error bars are obtained from bootstrapping with 70% confidence interval. *N* indicates the total number of molecules analyzed. (**D**) (Top) Schematic of λ-DNA containing three DSBs at different positions. Cy3 is labeled at each DSB. Biotin and digoxigenin (Dig) are modified at either end of λ-DNA. (Bottom) Schematic of DNA curtain for λ-DNA containing three DSBs. (**E**) DNA curtain image with λ-DNA containing three DSBs. Cy3 fluorescence puncta are well placed at the DSB locations that are denoted by arrowheads. “B” and “P” at the left indicate the barrier and pedestal positions, respectively. (**F**) Position distributions of Cy3 fluorescence puncta from Fig. 3E. The centers of Cy3 peaks are 11.2 ± 1.6, 21.8 ± 1.6, and 33.0 ± 1.8 kb from fitting with multiple Gaussian functions. The peak centers match well with the DSB insertion positions. Error bars are obtained from bootstrapping with 70% confidence interval. *N* indicates the total number of molecules analyzed.

The utility of this architecture was further demonstrated using dual-bead optical traps combined with confocal imaging, in which both ends of the damaged λ-DNA were tethered to optically trapped beads (Supplementary Fig. S5D). In this geometry, the position of Cy3 and Cy5 overlapped, and Cy5 fluorescence marked fully assembled internal DSBs (Supplementary Fig. S5E). Statistically, 12 of 19 molecules (63.2%) displayed both Cy3 and Cy5 signals, and 2 out of 19 molecules showed only Cy5 signals. Therefore, the effective assembly efficiency was estimated to be ∼73.7% (14 of 19 molecules), with a 95% confidence interval of 48.8%–90.9%, which is underestimated taking Cy5 photobleaching into consideration (Supplementary Fig. S5F). These results indicate that CGRID can generate internal DSBs in long DNA with high efficiency and in a format compatible with force-free single-molecule interrogation.

### Multiple internal DSBs can be introduced into a single DNA molecule

To test whether CGRID can scale beyond a single lesion, we generated λ-DNA molecules containing three blunt-ended internal DSBs labeled with Cy3 (Fig. 3D). DNA curtain imaging revealed three well-separated fluorescence puncta at the expected positions along individual DNA molecules (Fig. 3E). The spatial distribution showed three distinct peaks corresponding to the designed break sites (Fig. 3F). These data demonstrate that CGRID can install multiple DSBs within a single long DNA substrate. Moreover, because each insertion sequence can be independently designed, the multiple DSBs can be programmed to present distinct end configurations—including 5′ overhangs, 3′ overhangs, or blunt ends—within a single substrate. Hence, our approach increases experimental throughput and enables more complex substrate architectures for studying DSB repair.

### CGRID-generated internal DSBs are recognized and processed by pathway-relevant repair proteins

We next asked whether the engineered internal DSB substrates are competent for recognition and processing by DSB repair proteins. DSBs are repaired by homologous recombination and nonhomologous end joining (NHEJ) according to the cell cycle [14, 45, 46]. Besides these two major pathways, DSBs are also repaired by alternative end joining such as microhomology-mediated end joining (MMEJ) catalyzed by Polθ [45–47]. To model MMEJ, we designed an internal DSB carrying a 21-nt single-stranded overhang containing a 6-nt microhomology tract and a 15-nt poly (dT) segment (Fig. 4A and B). We purified a truncated human Polθ construct encompassing the polymerase domain and part of the linker region (Polθ_QM1) and monitored extension using DNA curtains (Supplementary Fig. S3A and B). In the presence of Polθ_QM1, dATP, and a Cy5-labeled complementary oligonucleotide, stable fluorescence accumulated at the internal DSB (Fig. 4C top and D). Binding of the Cy5-labeled oligomer to the internal DSB was confirmed by disappearance of the fluorescence signal when the flow was turned off (Fig. 4C middle). These data showed Polθ-dependent extension from the microhomology and stabilization of the annealed oligonucleotide. This stable signal was not observed in the absence of Polθ_QM1, indicating that productive engagement of the substrate depended on Polθ_QM1 activity (Fig. 4C bottom). These observations were consistent with bulk biochemical assays (Supplementary Fig. S3C).

**Figure 4.**
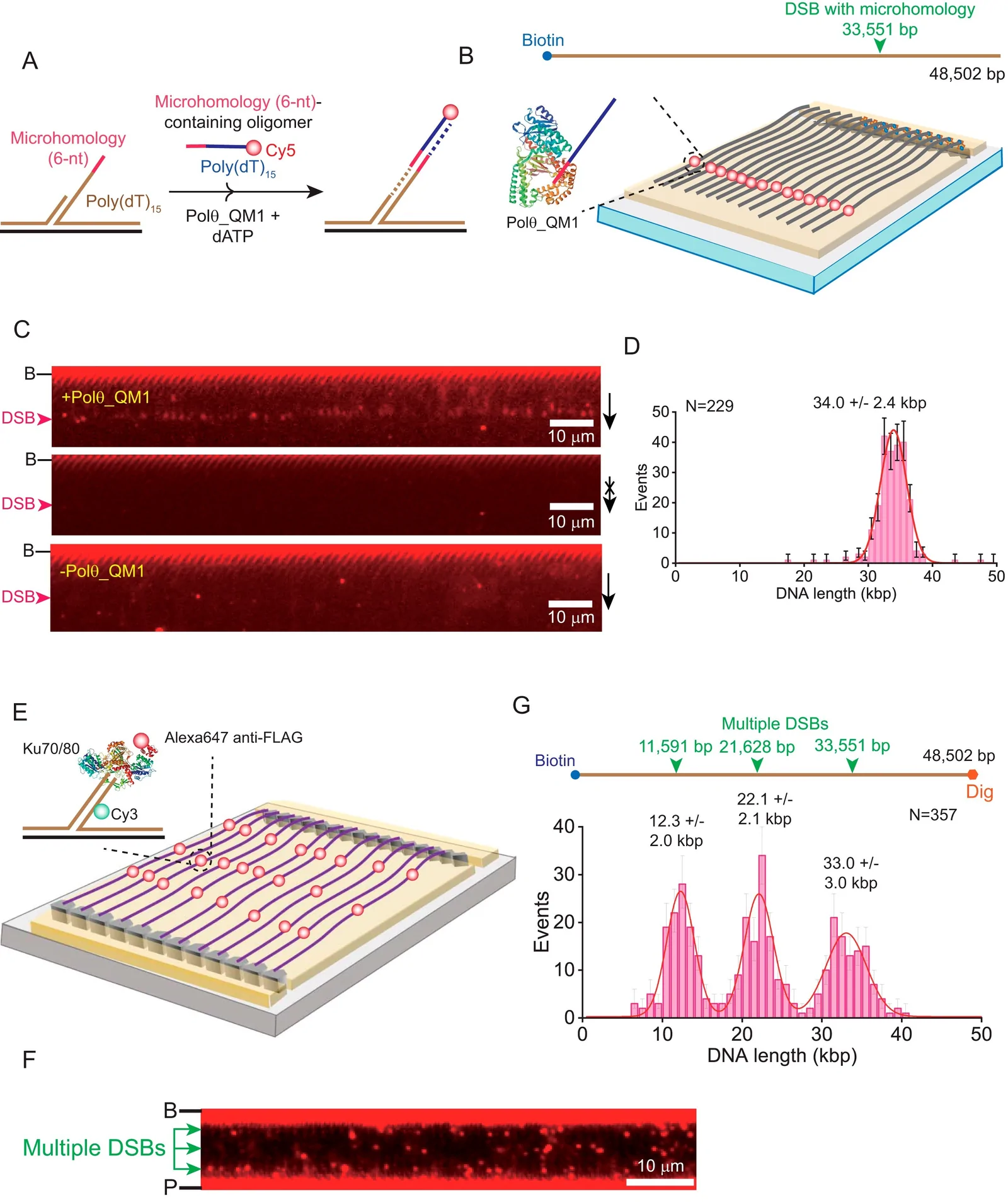
Direct visualization of interactions between DSB(s) and DSBR proteins. (**A**) Schematic of microhomology-mediated end joining (MMEJ) and DNA curtain. A DNA DSB with 3′ single-stranded DNA overhang is inserted into λ-DNA. When Polθ and Cy5-labeled oligomer with microhomology are incubated with the DSB, Polθ pairs the two microhomologies and extends DNA from them. Then, the Cy5-oligomer remains stable in λ-DNA. (**B**) Schematic of single-tethered DNA curtain for Polθ-mediated microhomology extension. When Polθ_QM1 elongates from the Cy5 oligomer at the microhomology, the Cy5 oligomer remains bound to DNA. The structure of Polθ_QM1 is generated using AlphaFold3. (**C**) (Top) DNA curtain image showing the stable binding of Cy5-oligomers by Polθ at the DSB site in the presence of buffer flow. (Middle) When the flow is turned off, DNA molecules recoil out of the evanescent field, and hence the Cy5 signals disappear, indicating that the Cy5-oligomers are bound to DNA. (Bottom) In the absence of Polθ, Cy5 oligomers with microhomology do not stably bind to DNA even under buffer flow. The arrowhead indicates the position of the DSB. The arrow at the right indicates the direction of buffer flow. The arrow with “X” indicates the absence of flow. (**D**) Binding distribution histogram of Cy5 in DNA curtains showing a single distinct peak, the center of which is 34.0 ± 2.4 kb from single Gaussian fitting. The peak corresponds to the location of the DSB position containing microhomology. *N* indicates the total number of molecules analyzed. Error bars are obtained from the bootstrapping with 70% confidence interval. (**E**) Schematic of double-tethered DNA curtain for NHEJ using λ-DNA containing three internal DSBs in Fig. 3D. Ku70/80, which is labeled with Alexa647 anti-FLAG antibody, recognizes DSBs. The structure of Ku70/80 is adopted from the Protein Data Bank (PDB ID: 1JEY). (**F**) DNA curtain image for Ku70/80 binding to internal DSBs. The DSB locations are denoted by arrowheads. “B” and “P” at the left indicate the barrier and pedestal positions, respectively. (**G**) Distribution of Alexa647 fluorescence puncta (Ku70/80) showing three distinct peaks. The peak centers are fitted with multiple Gaussian functions and match well with DSB insertion positions.

We then tested recognition of internal blunt-ended DSBs by human Ku70/80, an early sensor in classical NHEJ. Purified Ku70/80 complexes carrying an *N*-terminally FLAG-tagged Ku80 subunit showed the expected binding behavior in ensemble assays, consistent with previous studies (Supplementary Fig. S3D and E) [48, 49]. Ku70/80 was fluorescently visualized using an Alexa647-conjugated anti-FLAG antibody (Supplementary Fig. S3F). When introduced into double-tethered DNA curtains containing three internal blunt-ended DSBs, Ku70/80 bound preferentially at the engineered break positions (Fig. 4E and F). The binding distribution contained three discrete peaks coincident with the DSB sites (Fig. 4G). Optical trap experiments further showed direct association of Ku70/80 with an internal single DSB without detectable long-range diffusion, consistent with previous studies (Supplementary Fig. S3G) [17]. Together, these experiments show that CGRID-generated DSB substrates are competent for recognition by distinct repair factors and can distinguish different modes of DSB processing at the single-molecule level.

### CGRID supports efficient lesion insertion into nucleosomes

Studying how damage in nucleosomes and chromatin is repaired is important for understanding eukaryotic DNA repair mechanisms. To this end, we applied CGRID to Widom 601 DNA and examined whether modified DNA could be reconstituted into nucleosomes *in vitro* after lesion insertion. We purified recombinant *Xenopus laevis* histones, assembled histone octamers, and reconstituted mononucleosomes by salt dialysis [50]. We first inserted a fluorescein-modified oligonucleotide near SHL +3, where the modified base is expected to face the solvent rather than the histone octamer (Fig. 5A and B). Paired nicks on Widom 601 DNA were formed by sequential treatment of nCas9 complexes (Fig. 5C). To reduce off-target effects, competitor plasmid DNA was included during nicking. Denaturing gel analysis confirmed efficient and accurate nicking of Widom 601 DNA (Fig. 5D). Following gap replacement and ligation, fluorescein insertion efficiency was estimated to be ∼95% based on antibody-dependent mobility shifts of the modified DNA (Fig. 5E and F). The modified Widom 601 substrate was then reconstituted into mononucleosomes efficiently, with little or no detectable free DNA at optimal histone-to-DNA ratios (Fig. 5G). MNase digestion yielded the expected nucleosome-protected DNA fragments (100–150 bp), which retained fluorescein fluorescence but not the terminal Cy5 signal used for substrate preparation (Fig. 5H). Antibody supershift experiments further confirmed that fluorescein was retained after nucleosome assembly and remained solvent accessible (Fig. 5I). The nonshifted nucleosome band attributable to Widom 601 DNA without fluorescein was barely observed. The ratio between nonshifted and shifted band intensities indicated the fluorescein insertion efficiency, which was ∼96% (Fig. 5J). These data indicate that CGRID supports efficient and position-specific lesion installation in nucleosomal DNA without compromising Widom 601 sequence and nucleosome assembly.

**Figure 5.**
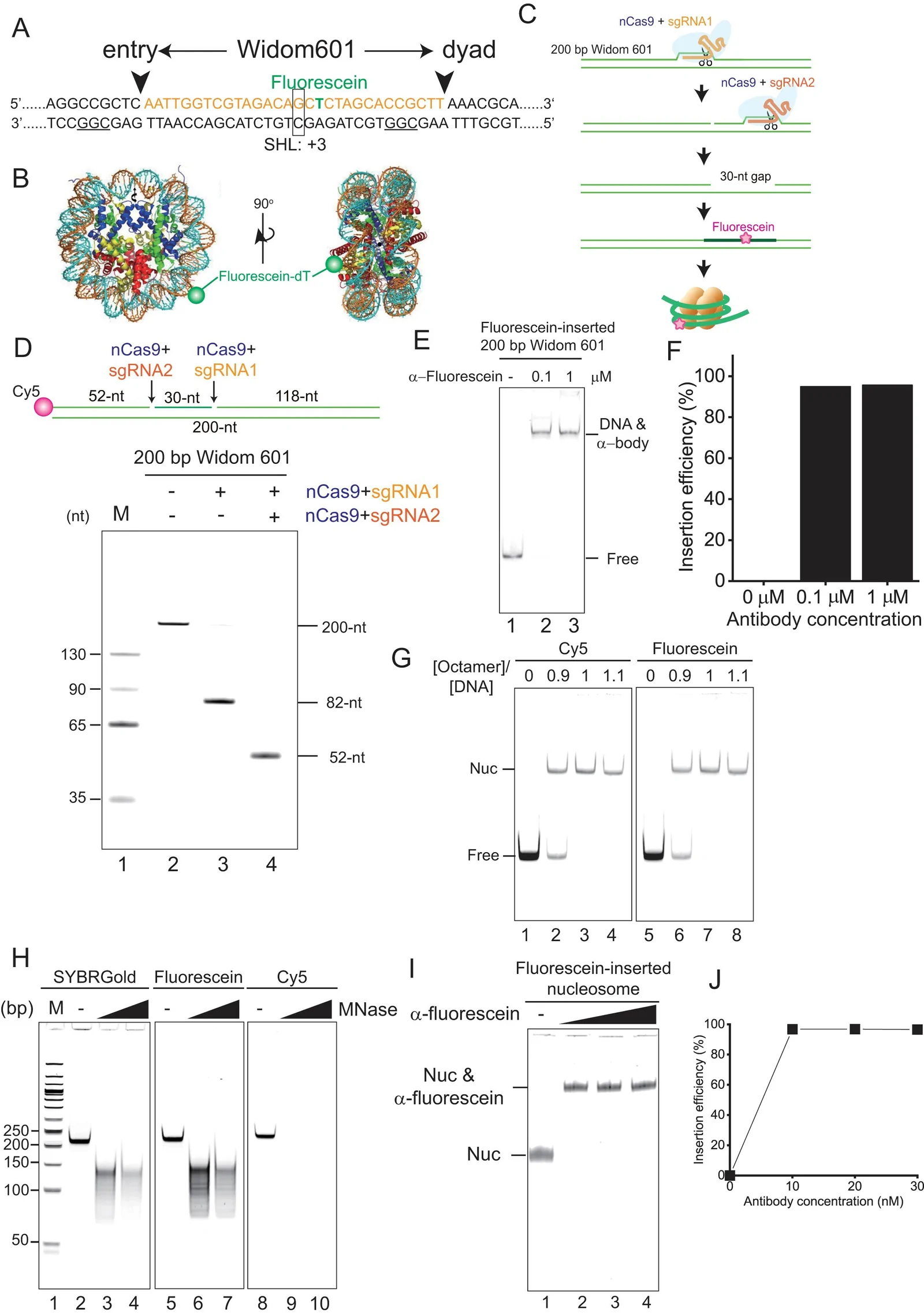
Lesion insertion into a nucleosome. (**A**) Fluorescein insertion position in Widom 601 sequence. The arrowheads indicate the cleavage sites by nCas9. The box denotes SHL: +3. (**B**) Structure of fluorescein-inserted nucleosome (PDB: 1KX5). The ball represents fluorescein in a nucleosome. (**C**) Strategy of fluorescein insertion into 200 bp Widom 601 DNA. Sequential treatment of two nCas9s generates a 30-nt gap in 200 bp Widom 601 DNA. After fluorescein-modified oligomer is hybridized and ligated, nucleosome is reconstituted. (**D**) (Top) Schematic of nCas9 treatment to 200 bp Widom 601 DNA. (Bottom) Denaturing gel for the nCas9-treated DNA. Sequential treatment produces DNA fragments of proper length, indicating that nCas9s completely make nicks on the DNA. (**E**) Gel shift assay for the fluorescein-inserted Widom 601 using antifluorescein antibody (α-fluorescein). The ratio of shifted and nonshifted bands indicates the fluorescein insertion efficiency. (**F**) Fluorescein insertion efficiency estimated by dividing the intensity of shifted band by sum of the intensities of shifted and nonshifted bands in Fig. 5E. (G) Gel shift assay for nucleosome reconstitution with fluorescein-inserted Widom 601 DNA. (**H**) MNase assay for nucleosomes with fluorescein-inserted Widom 601 DNA. (**I**) Supershift of fluorescein-containing nucleosome by α-fluorescein. (**J**) Quantification of fluorescein-insertion efficiency into a nucleosome. The fluorescein-insertion efficiency was estimated by dividing the shifted band (nucleosome + α-fluorescein) intensity by the intensity sum of shifted and unshifted (nucleosome only) bands.

### Lesion position within a nucleosome determines APE1 accessibility

To demonstrate the utility of CGRID for chromatin-based repair analysis, we inserted single AP sites at SHL +3, +3.5, or −6 in Widom 601 nucleosomes and measured incision by APE1 (Fig. 6A and B). These sites were selected to compare the effects of both translational position and rotational orientation: the lesion at SHL +3 is solvent-exposed, whereas the lesions at SHL +3.5 and −6 face the histone octamer, with the latter positioned near the entry–exit region (Fig. 6B).

**Figure 6.**
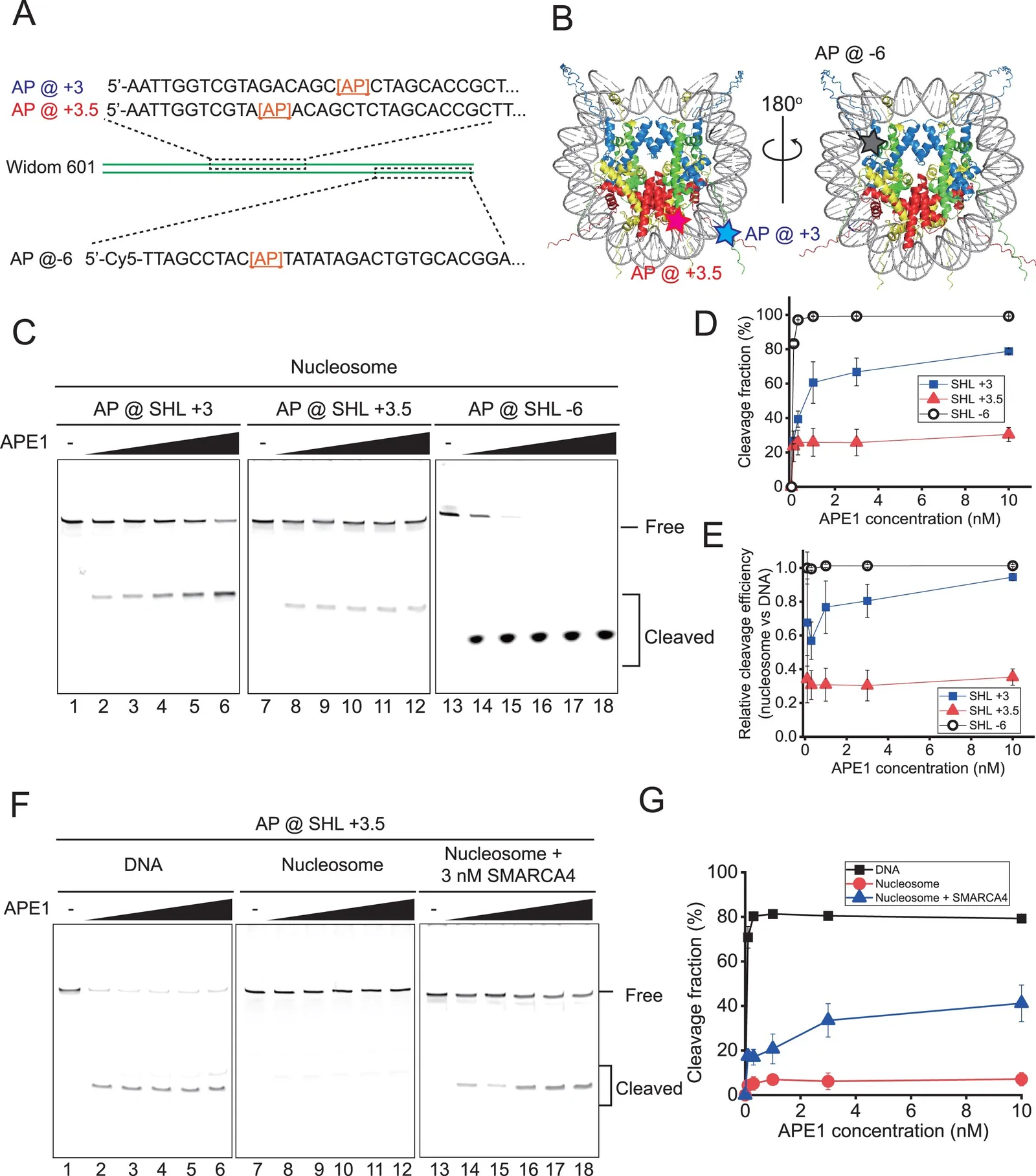
AP-site recognition and processing by APE1 within a nucleosome. (**A**) Schematic of AP-site positions at SHL +3, +3.5, and −6 in the 149 bp Widom 601 sequence. Cy5 is labeled at 5′ end. (**B**) Structural depiction of AP-site positions within a nucleosome. Blue, red, and black stars indicate AP sites at SHL +3, +3.5, and −6, respectively. Nucleosome structures are generated from AlphaFold3 simulation using the *Xenopus laevis* histone octamer and the 149 bp Widom 601 sequence. (**C**) Denaturing gel images showing APE1-mediated cleavage of AP sites in nucleosomes at different AP-site positions. (**D**) Quantification of the data shown in Fig. 6C. Errors are standard errors in triplicate. (**E**) Relative cleavage efficiency in nucleosomes compared with naked DNA at different AP-site positions. Errors are propagated from Fig. 6D and S6B. (**F**) Denaturing gel images showing APE1-mediated cleavage of AP sites at SHL +3.5 in naked DNA (200 bp Widom 601), nucleosomes, and nucleosomes incubated with 3 nM SMARCA4. (**G**) Quantification of the data shown in Fig. 6F. Errors are standard errors in triplicate.

In naked DNA, APE1 cleaved all three substrates efficiently, indicating that lesion position alone did not intrinsically impair enzyme activity. The ∼80% cleavage observed at SHL +3 and +3.5 in naked DNA was likely due to incomplete AP-site incorporation (Supplementary Fig. S6A and B). In nucleosomes, however, incision was strongly dependent on lesion position (Fig. 6C–E). Although cleavage at SHL +3 was reduced relative to naked DNA, it increased with higher concentrations of APE1 and eventually approached the level observed for free DNA. By contrast, the octamer-facing lesion at SHL +3.5 remained refractory to cleavage even at elevated enzyme concentrations (Fig. 6C and D). Notably, the lesion at SHL −6, despite facing the histone octamer, was cleaved with an efficiency comparable to that observed in naked DNA (Fig. 6C and D). When cleavage fractions were normalized to the corresponding naked DNA values, the positional dependence remained consistent (Fig. 6E). These results are consistent with a previous study showing that AP sites near the entry–exit region are more rapidly cleaved by APE1 than those near the dyad [51]. In addition, near the entry–exit region, solvent-exposed AP sites are cleaved more efficiently than histone-facing AP sites [51, 52]. Taken together, these data show that both rotational exposure and local nucleosome dynamics contribute to repair accessibility and illustrate how CGRID can be used to systematically probe lesion processing in chromatin contexts [51–54].

### A chromatin remodeler SMARCA4 promotes APE1 cleavage of AP sites in nucleosomes

Chromatin remodelers use ATP hydrolysis to reposition, slide, or evict histone octamers, thereby increasing the accessibility of DNA lesions to repair enzymes in chromatin. SWI/SNF family remodelers have been implicated in BER [55, 56]. We therefore asked whether SMARCA4, the catalytic ATPase subunit of BAF/SWI/SNF chromatin remodeling complexes, could facilitate APE1-mediated incision of AP sites embedded in nucleosomes [57]. Recombinant SMARCA4 repositioned histone octamers in the presence of ATP (Supplementary Fig. S6C). In the presence of SMARCA4, APE1 cleaved the solvent-exposed AP site at SHL +3 more efficiently than in its absence (Supplementary Fig. S6D and E). SMARCA4 also enhanced APE1-mediated incision at SHL +3.5, where the AP site faces the histone octamer and is poorly accessible to APE1 alone (Fig. 6F and G). Thus, SMARCA4-dependent chromatin remodeling overcomes the nucleosomal barrier to AP-site incision. These results establish CGRID as a platform for generating precisely positioned DNA lesions in nucleosomes and for quantitatively probing how chromatin remodelers regulate repair-enzyme access in a defined chromatin context.

## Discussion

CGRID addresses a central technical limitation in DNA repair research: the lack of a general, programmable, and efficient strategy for installing defined lesions at arbitrary positions in long DNA and chromatin substrates. Existing methods have enabled important mechanistic advances, but many depend on pre-engineered nicking sites, repeated sequence cassettes, or labor-intensive substrate assembly, thereby limiting positional freedom, lesion diversity, or scalability. By coupling paired nCas9s-mediated gap formation to replacement with synthetic lesion-containing oligonucleotides, CGRID converts lesion installation into a modular design problem specified largely by sgRNA choice.

A key strength of the method is its compatibility with long DNA substrates used in single-molecule assays. In λ-DNA, CGRID allowed efficient installation of a single AP site at a user-defined internal position and supported quantitative analysis of APE1 target search using double-tethered DNA curtains (Fig. 2). This is important because previous single-molecule studies often relied on multiple lesions or lesions restricted to predefined positions. By enabling analysis of a single lesion in long DNA, CGRID improves control over lesion number and position and facilitates more direct interpretation of search and recognition behavior.

The ability to generate internal DSBs is another important advance. In many single-molecule configurations, access to a DNA end and maintenance of DNA extension are coupled, making it difficult to study DSB repair without applying continuous hydrodynamic force. CGRID uncouples these constraints by allowing structurally defined DSBs to be positioned internally within double-tethered DNA molecules. This architecture enabled direct visualization of Ku70/80 engagement and Polθ-dependent processing in substrates that remain extended even in the absence of flow (Fig. 4). The successful installation of multiple internal DSBs further indicates that the method is scalable and should be useful for increasing event counts and for building more complex repair substrates.

CGRID can be also applied to insert DNA lesions into plasmid DNA for *in vitro* experiments [58, 59]. However, the nicks by nCas9 would relax plasmid supercoiling, altering the topological state of the substrate. If lesion-containing plasmids were transformed into cells, the lesions would be repaired by endogenous DNA repair pathways.

CGRID also extends programmable lesion engineering into nucleosomal DNA. In conventional nucleosome-based lesion studies, changing lesion position often requires rebuilding the substrate through new ligation or synthesis schemes. In contrast, CGRID allows lesion repositioning by redesigning sgRNAs while preserving DNA sequences. This feature makes systematic comparisons of lesion accessibility within a common nucleosome template substantially more practical. Our APE1 incision experiments illustrate the power of this approach by showing that lesion processing depends strongly on both rotational setting and position within the nucleosome (Fig. 6D). In particular, lesions at SHL +3.5 that face the histone octamer remain poorly accessible, whereas lesions near the entry–exit region can remain permissive to repair despite inward orientation, consistent with enhanced local DNA breathing at the nucleosome edge [60, 61]. These findings are consistent with the previous study [26].

Importantly, CGRID further enables defined chromatin substrates to be coupled with chromatin-remodeling reactions. By placing AP sites at specific SHLs, we found that SMARCA4, the catalytic ATPase subunit of BAF/SWI/SNF chromatin remodeling complexes, enhanced APE1-mediated incision of nucleosomal AP sites. This effect was observed not only at a solvent-exposed lesion but also at an octamer-facing lesion that was otherwise poorly accessible to APE1 (Fig. 6G and Supplementary Fig. S6E). These results support a model in which SMARCA4-dependent remodeling partially relieves the nucleosomal barrier to BER by increasing local lesion accessibility. More broadly, they show that CGRID can be used not only to map the intrinsic effects of nucleosome geometry on repair, but also to dissect how chromatin remodelers, histone variants, or chromatin-associated factors dynamically regulate lesion processing in a defined chromatin context.

The present study focused on AP sites and DSB-like structures, but the underlying design principle is broader. Any lesion or DNA architecture that can be encoded within a replacement oligonucleotide should in principle be compatible with the method. CGRID should therefore be adaptable to a wider range of DNA damage substrates for studying base excision repair, nucleotide excision repair, mismatch repair, end joining pathways, and replication-associated damage processing. More broadly, the method bridges a persistent gap between substrate generation and mechanistic interrogation by enabling the same programmable lesion-engineering framework to be used across biochemical assays, single-molecule imaging, and chromatin-based experiments.

## Supplementary Material

gkag965_Supplemental_File

## Data Availability

The data underlying this article are available in the article and in its online Supplementary Data. Additional raw microscopy data are available from the corresponding author upon reasonable request.
