# Supplementary material for: CRISPR-mediated gap-based replacement enables site-specific DNA lesion insertion into long DNA and nucleosomes
Source: Nucleic Acids Res. 2026 Oct 13;54(19):gkag965. doi: 10.1093/nar/gkag965 (PMC13659233; doi:10.1093/nar/gkag965)
Supplement: Supplementary file 1 [file gkag965_supplemental_file.docx]

**Supplementary Information**

**CRISPR-mediated gap-based replacement enables site-specific DNA lesion insertion into long DNA and nucleosomes**

Youngseo Kim^1†^, Soyeong An^1†^, Jimin Choi^1^, Moonjung Choi^2^, Gyeongjin Lee^1^, Kei-ichi Takata^2,3^, Roopesh Anand^4^, Kyungjae Myung^2,3^, and Ja Yil Lee^1,2 *^

^1^ Department of biological sciences, Ulsan National Institute of Science and Technology, Ulsan 44919, Republic of Korea

^2^ Institute for Basic Science Center for Genomic Integrity, Ulsan 44919, Republic of Korea

^3^ Department of biomedical engineering, Ulsan National Institute of Science and Technology, Ulsan 44919, Republic of Korea

^4^ Institute of Molecular Biology (IMB), Mainz 55128, Germany

^†^ Equally contributed

* Correspondence: Ja Yil Lee, [biojayil@unist.ac.kr](mailto:biojayil@unist.ac.kr)

**Key words:** CRISPR-Cas9 nickase, DNA lesion insertion, single-molecule imaging, damage-containing nucleosome, DNA repair

**Running title:** CRISPR-mediated damage insertion into DNA

**Contents**

**I. Supplementary Materials and Methods**

**1. DNA preparation**

*1-1. Preparation of DNA fragments for damage insertion using CRISPR-Cas9 nickase*

*1-2. Preparation of* λ-*DNA for DNA curtains*

*1-3. Preparation of* λ-*DNA for dual-trap optical tweezers*

**2. Protein preparation**

*2-1. Purification of Cas9 D10A nickase*

*2-2. Human Ku70/80 purification and activity test*

*2-3. Polθ_QM1 purification and activity test*

*2-4. APE1 and APE1_D210N activity test*

**3. Histone purification and in vitro nucleosome reconstitution**

3-1. *Histone purification*

*3-2. Histone octamer assembly and purification*

*3-3. In vitro nucleosome reconstitution*

*3-4. Micrococcal nuclease (MNase) assay*

**4. DNA curtain assay**

*4-1. DNA curtain system*

*4-2. DNA curtain assay for Polθ_QM1*

*4-3. DNA curtain assay for Ku70/80*

*4-4. DNA curtain assay for APE1_D210N*

**5. DNA curtain data analysis**

*5-1. Position distribution of fluorescence signal*

*5-2. Calculation of diffusion coefficients of APE1_D210N*

**6. Dual-bead optical trap assays**

**II. Supplementary Tables**

**1. Table 1**. List of synthesized oligomers

**2. Table 2**. List of sgRNAs and IVT primers

**3. Table 3**. Composition of imaging buffer

**III. Supplementary Figures**

**1. Figure S1.** Preparation of nCas9 and single-guide RNA (sgRNA)

**2. Figure S2.** APE1 purification and activity test

**3. Figure S3.** Purification of Ku70/80 and Polθ and activity test

**4. Figure S4.** Position change of a Cy5-labeled oligonucleotide insertion in λ-DNA

**5. Figure S5.** Insertion of a DNA double-strand break (DSB)

**6. Figure S6.** SMARCA4 effect on APE1 cleavage of the AP site in a nucleosome

**IV. Supplementary References**

**I. Supplementary Materials and Methods**

***1. DNA preparation***

*1-1. Preparation of DNA fragments for damage insertion using CRISPR-Cas9 nickase*

To test the efficiency of DNA damage insertion, a 1,036 bp DNA fragment was generated via PCR amplification using lambda phage DNA (λ-DNA) (New England Biolabs, N3011S) as a template with primers (1036F and 1036R) (Supplementary Table 1). To insert a lesion into Widom 601 DNA, a plasmid containing Widom 601 sequence was provided from Professor Hajin Kim at Ulsan National Institute of Science and Technology, South Korea. 149 bp and 200 bp DNA fragments having a Widom 601 sequence were generated via PCR using the plasmid as a template with primers (149 bp: CY5-149 bp-F (149 bp-F) and CY5-149 bp-R (or 149 bp-R)); 200 bp: CY5-200 bp-F and 200 bp-R), respectively (Supplementary Table 1). All PCR products were purified by Expin™ PCR SV Kit (Gene All, 103-102).

*1-2. Preparation of* λ-*DNA for DNA curtains*

9 nM λ-DNA (New England Biolabs, N3011L) was mixed with 500-fold excessive oligomers containing biotin or digoxigenin (Lambda biotin-L/Lambda dig-R or Lambda biotin-R/Lambda dig-L) in 1× T4 ligase buffer (50 mM Tris-HCl [7.5], 10 mM MgCl₂, 10 mM DTT, and 1 mM ATP) (Supplementary Table 1). The mixture was heated up to 65°C for 20 minutes and subsequently cooled down to 23°C slowly for annealing. The oligomers were ligated to λ-DNA with T4 DNA ligase (Enzynomics, M001), which was then heat-inactivated at 65°C for 20 minutes. The λ-DNA was purified with Illustra MicroSpin^TM^ S-400 spin column (GE Healthcare, 27514001).

*1-3. Preparation of* λ-*DNA for dual-trap optical tweezers*

For the dual-trap optical tweezers, both ends of λ-DNA were multiply biotinylated. 8 nM of λ-DNA was mixed with 50 μM each of dATP, dTTP, and dGTP (Enzynomics, N002S), and Biotin-11-dCTP (Lumiprobe, 2715-1 μmol) in 1× NEBuffer™ 2 (New England Biolabs, B7002S). The mixture was heated at 65 °C for 20 minutes and immediately placed on ice. Subsequently, 15 units of Klenow Fragment (3’→5’ exo–) (New England Biolabs, M0212L) were added and incubated at 25 °C for 1 hour. Sodium acetate to a final concentration of 0.3 M was then added, followed by glycogen to a final concentration of 1 μg/μL. 100% ethanol was added at 2.5 times the sample volume and then incubated at –20 °C for 6 hours. The reaction was centrifuged at 20,000 g for 5 minutes, and the supernatant was removed. The pellet was washed with 70% ethanol and centrifuged again at 20,000 g for 5 minutes. Finally, the pellet was air-dried and resuspended in 1× TE buffer (10 mM Tris-HCl [8.0] and 0.1 mM EDTA).

**2. Protein preparation**

*2-1. Purification of Cas9 D10A nickase*

Cas9 D10A nickase (nCas9) was purified by following the previous protocol with slight modifications(1). nCas9 with 6x His and 1x HA tags at the amino terminus was cloned into pET28a plasmid vector. The plasmid containing nCas9 was transformed into *E. coli* BL21(DE3) strain. 1 L of *E. coli* cells were grown at 37°C in LB media supplemented with 50 μg/ mL kanamycin until OD_600_ reached ~0.4. The temperature then cooled down to 25°C and then cells were further grown until OD_600_ reached ~0.6, where the protein was expressed with 0.5 mM IPTG (isopropyl β-D-1-thiogalactopyranoside) at 25°C for 4 hours. Cells were harvested by centrifugation at 5,000 g and 18°C for 18 minutes. The cell pellet was resuspended in 100 mL of nCas9 lysis buffer (50 mM NaH_2_PO_4_ [8.0], 300 mM NaCl, 10 mM imidazole, 10 % glycerol, 1 mM DTT, HALT Protease Inhibitor Cocktail (Thermo Fisher Scientific, 78438), and 1 mM PMSF (Phenylmethanesulfonyl fluoride)). Cells were lysed by two-step sonication (Q700, Qsonica). Sonication was first applied at 30% amplitude with total on time 1 minute (10 seconds pulse on and 15 seconds pulse off), and then the total on time was changed to 10 minutes (1 second pulse on and 10 seconds pulse off). Cell lysates were clarified by ultracentrifugation at 100,000 g and 4°C for 30 minutes. The clarified lysates were loaded onto 5 mL of HisPur™ Ni-NTA Resin (Thermo Fisher Scientific, 88222), which was pre-equilibrated with nCas9 lysis buffer. Unbound proteins were washed out twice with 50 mL of Ni-NTA wash buffer (50 mM NaH_2_PO_4_ [8.0], 300 mM NaCl, 20 mM imidazole, and 1 mM DTT). nCas9 was eluted after 30-minute incubation in 30 mL of Ni-NTA elution buffer (50 mM NaH_2_PO_4_ [8.0], 300 mM NaCl, 250 mM imidazole, 10 % glycerol, and 1 mM DTT). Glycerol was added to the protein sample up to 16%, and then the sample was dialyzed against 1 L of nCas9 heparin A buffer (50 mM NaH_2_PO_4_ [8.0], 10 % Glycerol, and 1 mM DTT) at 4°C using Spectra/Por™ 4 dialysis tubing (12-14 kDa MWCO, Spectra/Por, 132706). The nCas9 heparin A buffer was exchanged after 2 hours and again after 1.5 hours. Dialyzed sample was then loaded onto HiTrap™ Heparin HP 5 mL column (Cytiva, 17040701), which was pre-equilibrated in nCas9 heparin A buffer. The proteins were eluted around 0.6 M NaCl during salt gradient from nCas9 heparin A buffer to nCas9 heparin B buffer (50 mM NaH_2_PO_4_ [8.0], 1 M NaCl, 10 % glycerol, and 1 mM DTT). Finally, glycerol was added to the protein sample up to 16%, and then the sample was dialyzed against 1 L of nCas9 storage buffer (20 mM HEPES-NaOH [7.5], 150 mM KCl, 10 % glycerol, and 1 mM DTT) at 4°C using Spectra/Por™ 4 dialysis tubing (12-14 kDa MWCO, Spectra/Por, 132706). The storage buffer was exchanged after 2 hours and again after 16 hours. The final sample was snap-frozen in liquid nitrogen and then stored at -80°C until use.

*2-2. Human Ku70/80 purification and activity test*

Human Ku70/80 was purified by the previous protocol with slight modifications (2). Ku70 without any tag and Ku80 with 6x His and 3x FLAG tag at the amino terminus were cloned into pET14b plasmid vector. The plasmid was transformed into *E. coli* Rosetta2 (DE3) pLysS strain. 2 L of *E. coli* cells were grown at 37°C in LB media supplemented with 33 μg/mL ampicillin until OD_600_ reached ~0.6. Protein was expressed by adding 0.25 mM IPTG, followed by further incubation at 37 °C for 1 hour. The culture was then shifted to 16°C and incubated for an additional 16 hours. Cells were harvested by centrifugation at 8,000 g and 4°C for 15 minutes. Cell pellet was resuspended in 70 mL Ku lysis buffer (50 mM Tris-HCl [8.0], 1 M NaCl, 5 % Glycerol, 0.4 M ammonium acetate, 10 mM imidazole, 2 mM 2-mercaptoethanol, 1 mM PMSF, 1x cOmplete EDTA-free Protease Inhibitor Cocktail (Roche, 11873580001), and 0.2 mg/mL lysozyme). Cells were lysed by 30% amplitude sonication (Q700, Qsonica) with total 5 minutes on time (5 seconds pulse on and 1 minute pulse off). Ultracentrifugation at 30,000 g and 4°C for 60 minutes clarified the cell lysates. The clarified lysates were incubated with 10 mL of TALON Superflow Metal Affinity Resin (Takara, 635670), which was pre-equilibrated with TALON wash buffer (50 mM Tris-HCl [8.0], 50 mM NaCl, 20% glycerol, and 1 mM PMSF), with 30-minute incubation. The resin was then washed with 300 mL of TALON wash buffer and transferred to a gravity column. Bound proteins were eluted with 50 mL of TALON elution buffer (50 mM Tris-HCl [8.0], 50 mM NaCl, 20% glycerol, 1 mM PMSF, and 500 mM imidazole). The eluted Ku70/80 was further purified by Superdex 200 Increase 10/300 GL column (Cytiva, 28-9909-44), which was pre-equilibrated in SEC buffer (50 mM Tris-HCl [8.0], 50 mM NaCl, 10% glycerol, 0.5 mM EDTA, 1 mM DTT, and 1 mM PMSF). Final eluates were loaded onto Mono Q® 5/50 GL column (Cytiva, 17-5166-01) pre-equilibrated with Mono Q wash buffer (50 mM Tris-HCl [8.0], 50 mM NaCl, 5 % glycerol, 0.5 mM EDTA, 1 mM DTT, and 1 mM PMSF). After the column was washed with 10 mL of Mono Q wash buffer, proteins were eluted by salt gradient from Mono Q wash buffer to Mono Q elution buffer (50 mM Tris-HCl [8.0], 1 M NaCl, 5 % glycerol, 0.5 mM EDTA, 1 mM DTT, and 1 mM PMSF). The final eluates were sequentially dialyzed against Ku dialysis buffer (50 mM Tris-HCl [8.0], 20% glycerol, 0.5 mM EDTA, 1 mM DTT and 1 mM PMSF) containing 200 mM, and 50 mM NaCl for 3 hours each, using Spectra/Por™ 4 dialysis tubing (12–14 kDa MWCO, Spectra/Por, 132706). The final samples were concentrated with Amicon (MWCO: 50 kDa) and were snap-frozen in liquid nitrogen followed by -80°C storage until use.

The activity of purified Ku70/80 complexes was tested by binding to DNA ends. 5 nM Cy5-labeled 66 bp duplex DNA (Ku70/80_Cy5 and Ku70/80_comp) was incubated with purified Ku70/80 complexes at different concentrations in 50 mM Tris–HCl [7.5], 100 mM KCl, 5% glycerol, 100 µg/mL BSA, and 1 mM DTT at 23°C for 30 minutes (Supplementary Table 1). The incubation was done in the dark to avoid Cy5 photobleaching. The reactions were resolved by 5% non-denaturing polyacrylamide gel electrophoresis (PAGE) in 0.5× TBE at 4°C. The gel was imaged using a Typhoon RGB scanner (Cytiva).

The binding between Ku70/80 complex and anti-FLAG antibody was assessed by DNA supershift. 5 nM Cy5-labeled 66 bp duplex DNA was incubated with 15 nM Ku70/80 complex in a reaction buffer containing 50 mM Tris–HCl [7.5], 100 mM KCl, 5% glycerol, 100 µg/mL BSA, and 1 mM DTT at 23°C for 30 minutes. After the initial incubation, anti-FLAG antibody (Sigma-Aldrich, F3165-1MG) was added at different concentrations (3, 30, and 300 nM), followed by an additional 30-minute incubation at 23°C. The reactions were analyzed on a 5% native polyacrylamide gel in 0.5× TBE at 4°C, which was imaged using a Typhoon RGB scanner (Cytiva).

*2-3. Polθ_QM1 purification and activity test*

A plasmid Sumo3-PolQM1 containing a truncated version of the human *POLQ* enzyme encompassing residues 1792–2590 (Polθ_QM1) was obtained from Addgene (Addgene, #78462) (3). The 6×His-SUMO tag in Polθ_QM1-pE-SUMO was replaced with a 10×His-GST tag via In-Fusion cloning (Takara, 638943) using the following primers: 10×His-GST for QM1 IFCF: AGATATACCATGGGTCATCATCATCATCATCATCATCATCATCAC, 10×His-GST for QM1 IFCR: GTTGTCTTTGAACCCGGGCCCCTGGAACAGAACTT (used to amplify the GST sequence from the GST-REV3L plasmid) , and QM1 for 10×His-GST IFCF: GGGTTCAAAGACAACAGCCCTATT, QM1 for 10×His-GST IFCR: ACCCATGGTATATCTCCTTCTTAAAG (used to amplify the QM1 sequence from Sumo3- Polθ_QM1) (4). Amplifications were performed using KOD Xtreme Hot Start DNA Polymerase (Sigma-Aldrich, 71975-M).

The recombinant protein was expressed following a previously described protocol (5). The plasmid was transformed into E. coli Rosetta 2 (DE3) pLysS competent cells (Novagen,71403). A single colony was inoculated into 5 mL of Luria–Bertani (LB) medium supplemented with 40 mM glucose, 50 µg/mL carbenicillin, and 50 µg/mL chloramphenicol, and cultured overnight at 37 °C. The overnight culture (1 mL) was used to inoculate 1 L of Terrific Broth (TB) medium supplemented with 25 mL of 50× 5032 sugar mix (25% glycerol, 2.5% glucose, 10% α-lactose monohydrate), 50 µg/mL carbenicillin, and 50 µg/mL chloramphenicol. A total of four 1-L cultures were prepared.

Cells grown at 20 °C for 60 hours in a shaking incubator at 170 rpm were harvested by centrifugation (4000 g, 20 minutes) and washed once with ice-cold phosphate-buffered saline (PBS). Following an additional centrifugation step (4000 g, 20 minutes), the cell pellet was resuspended in five volumes of equilibration buffer (50 mM HEPES-KOH [8.0], 300 mM NaCl, 5 mM CaCl₂, 20 mM imidazole, 10% glycerol, 1.5% NP-40, 5 mM β-mercaptoethanol, 100 µg/ml lysozyme, and EDTA-free protease inhibitor mixture (Roche, 04693132001)). The resuspended cells were sonicated on ice using 20 cycles of 10 seconds pulses with 20 seconds intervals at 20% amplitude. The crude extract was clarified by two rounds of centrifugation at 27,000 g for 35 minutes at 4 °C to remove precipitates. The resulting supernatant (~700 mL) was then incubated overnight at 4 °C with 5 mL of His-Tag Purification Resin (Roche, 08778850001) in a beaker with gentle stirring using a stir bar. The mixture was transferred to an Econo-Pac chromatography column (Bio-Rad, 7321010), and the resin was washed with 15 mL of Ni wash buffer (50 mM HEPES-KOH [8.0], 300 mM NaCl, 20 mM imidazole, 10% glycerol, 0.005% NP-40, 5 mM β-mercaptoethanol, and EDTA-free protease inhibitor mixture (Roche, 04693132001)) three times. The protein was eluted from the resin using 10 mL of elution buffer consisting of 50 mM Tris-HCl [7.5], 400 mM NaCl, 1 mM EDTA, 10% glycerol, 0.01% NP-40, 2 mM DTT, 5 mM β-mercaptoethanol, 250 mM imidazole, and an EDTA-free protease inhibitor cocktail (Roche Applied Science).

The eluted fraction (10 mL) was incubated with 1 mL of Glutathione Sepharose 4B at 4 °C for 30 minutes in a 15-mL tube. The mixture was transferred to an Econo-Pac chromatography column (Bio-Rad, 7321010), washed three times with 5 mL of 0.4 M NaCl TEMG buffer (50 mM Tris-HCl [7.5], 400 mM NaCl, 1 mM EDTA, 10% glycerol, 0.01% NP-40, 2 mM DTT, 5 mM β-mercaptoethanol, 250 mM imidazole, and an EDTA-free protease inhibitor cocktail (Roche, 04693132001)), and subsequently washed three times with 5 mL of 1 M NaCl TEMG buffer of the same composition, except containing 1 M NaCl instead of 400 mM. The bound protein was eluted with 3 mL of GST elution buffer (50 mM Tris-HCl [7.9], 400 mM NaCl, 1 mM EDTA, 10% glycerol, 0.01% NP-40, 2 mM DTT, 5 mM β-mercaptoethanol, 10 mM reduced glutathione, and an EDTA-free protease inhibitor cocktail). To exchange the buffer, the 3 mL eluate was applied to an Econo-Pac 10DG column and replaced with PreScission cleavage buffer (50 mM Tris-HCl [pH 6.8], 150 mM NaCl, 1 mM DTT, 1 mM EDTA, and 0.01% NP-40), yielding a final volume of 4 mL. The His-GST tag was then cleaved by adding 20 µL of PreScission Protease and incubating at 4 °C for 4 hours. To remove the cleaved His-GST tag, the reaction mixture was passed through 500 µL of His-Tag Purification Resin followed by 500 µL of Glutathione Sepharose 4B, and the flow-through fraction was collected.

The fraction was loaded onto a Mono S 5/50 GL column (Cytiva, 17516801), washed with 20 mL of 150 mM FPLC buffer (50 mM HEPES-KOH [8.0], 10% glycerol, 150 mM NaCl, 5 mM β-mercaptoethanol, and 0.005% NP-40), and eluted with a 10 mL gradient from the 150 mM buffer to the 2 M FPLC buffer (50 mM HEPES-KOH [8.0], 10% glycerol, 2 M NaCl, 5 mM β-mercaptoethanol, and 0.005% NP-40). The target protein eluted at approximately 440 mM NaCl. The eluted fractions were pooled, and the buffer was exchanged to 150 mM FPLC buffer using an Econo-Pac 10DG column (Bio-Rad, 7322010). The sample was then applied to a HiTrap Heparin HP column (Cytiva, 17040701), washed with 20 mL of 150 mM FPLC buffer, and eluted with a 10 mL gradient from the 150 mM buffer to the 2 M FPLC buffer. The protein eluted at approximately 340 mM NaCl. The final fractions were buffer-exchanged into POLQ dilution buffer (37.5 mM Tris-HCl [8.0], 40 mM NaCl, 6.25% glycerol, 2.5 mM DTT, and 0.01% Triton X-100) using an Econo-Pac 10DG column, concentrated with an Amicon Ultra-0.5 mL 50K centrifugal filter unit (Millipore, UFC5050), and stored at -80 °C.

The enzymatic activity of purified Polθ_QM1 was tested by an elongation assay. 0, 10, 20, and 40 nM Polθ_QM1 were incubated with 100 nM Insert_polθ_3 oligomer and 100 nM Insert_polθ_4 oligomer at different concentrations in Polθ reaction buffer (30 mM Tris-HCl [8.0], 6.4 mM NaCl, 10 mM MgCl₂, 0.4 mM DTT, 0.2 mg/mL BSA, 0.01% Triton X-100, 0.5% glycerol, and 5 µM dATP) at 37°C for 20 minutes. The reactions were analyzed by 7 M urea denaturing 20% PAGE in 1× TBE buffer at 23°C for 5 hours. Then the gel was imaged using a Typhoon RGB scanner (Cytiva).

*2-4. APE1 and APE1_D210N activity test*

To test the cleavage of AP-containing DNA by purified wild-type APE1 and APE1_D210N, AP_25_Cy5 oligomer and AP_25_comp oligomer were hybridized. 10 nM Cy5-labeled AP-containing DNA was incubated with wild-type APE1 or APE1_D210N at different concentrations (0, 3, 10, 30, and 100 nM) in 20 mM Tris-HCl [7.5], 50 mM NaCl, 5 mM MgCl_2_, 1 mM DTT, and 0.1 mg/ml BSA at 23°C for 10 minutes. The reaction was stopped by adding an equal volume of stop solution containing 95% formamide and 10 mM EDTA. The mixtures were heated at 95°C for 10 minutes to denature all DNA oligomers. The reactions were analyzed by 8 M urea denaturing 15% PAGE in 1x TBE buffer at 23°C for 1 hour. The gel was imaged using a Typhoon RGB scanner (Cytiva).

**3. Histone purification and in vitro nucleosome reconstitution**

3-1. *Histone purification*

Canonical histones (H2A, H2B, H3, and H4) of *Xenopus laevis* were individually purified based on a previous paper (6). Plasmids containing each histone were transformed into BL21(DE3)pLysS *E.coli* strain (Invitrogen, C6060-03). Single colony was inoculated into 200 mL LB media supplemented with ampicillin and then grown in 37°C shaking incubator at 225 rpm overnight. This pre-culture was inoculated again into 8 L of LB media. The cells were grown in 37°C shaking incubator until OD600 reached about 0.6. Each histone protein was induced by 0.4 mM IPTG and then overexpressed at 37°C for 3 hours. Subsequently, cells were harvested at 5,316 g and 18°C for 20 minutes. Cell pellets were resuspended in 30 mL wash buffer A (50 mM Tris-HCl [7.5], 100 mM NaCl, and 1 mM 2-mercaptoethanol). Approximately 60 mg of lysozyme (Sigma, L6876) was added into the resuspended cells and then incubated at 37°C for 7 minutes. Then wash buffer B (50 mM Tris-HCl [7.5], 100 mM NaCl, 1 mM 2-mercaptoethanol, and 1 mM PMSF) was added up to 100 mL. The cell extracts were blended on ice using a homogenizer (IKA, T 10 basic ULTRA-TURRAX). To avoid cells from heating, cells were blended for only 5 seconds and cooled on ice for 5 seconds. These blending and icing steps were repeated 6 times (total 1 minute). Blended cell extracts were spun down at 18,000 g at 4°C for 20 minutes for inclusion body preparation. During inclusion body preparation, all supernatants were saved to check target proteins were not in the supernatants. The first supernatant was kept, and pellets were resuspended in 75 mL wash buffer B supplemented with 1% Triton X-100 using the homogenizer just 3-4 times every five seconds. Centrifugation was repeated at 18,000 g at 4°C for 15 minutes. The second supernatant was removed and saved, and pellets were resuspended in 75 mL wash buffer B supplemented with 1% Triton X-100 by pipetting. Resuspended cells were spun down in the same condition. Third supernatant was removed and saved, and pellets were resuspended in 75 mL wash buffer B without Triton X-100 by pipetting. Resuspended cells were pelleted at 18,000 g at 4°C for 10 minutes. Fourth supernatant was removed and saved, and pellets were resuspended in 75 mL wash buffer B without Triton X-100 by pipetting. Resuspended cells were pelleted down at 18,000 g at 4°C for 10 minutes. Fifth supernatant was removed and saved. Pellets were resuspended in 2~3 mL DMSO for 30 minutes. 20 mL unfolding buffer (6 M Guanidinium HCl, 20 mM Sodium Acetate [5.2], and 5 mM DTT) was added and rotated until pellets were fully resuspended. The resuspended pellets were spun at 14,000 g at 23°C for 10 minutes, and supernatant was saved. Pellet was resuspended again in 10 mL unfolding buffer and spun in the same condition. The two supernatants in unfolding buffer were combined and loaded onto S-200 column (GE Healthcare, Sephacryl S-200 High Resolution), which was equilibrated with SAUDE200 buffer (7 M urea, 20 mM Sodium Acetate [5.2], 5 mM 2-mercaptoethanol, 1 mM EDTA, 200 mM NaCl, and approximately 10 g Dowex Marathon MR-3 hydrogen and hydroxide form), using a peristaltic pump at 3 mL/min. After loading was finished, 10 mL fractions were taken at 0.5 mL/min in SAUDE200 buffer. All peaks were checked by 18% SDS-PAGE, and fractions containing histone protein were pooled and loaded at TSK-SP-5PW column (Merck, 816093), which was equilibrated with SAUDE200 buffer, at 3 mL/min. The sample was eluted by salt gradient from 0 M to 1 M NaCl in SAUDE buffer (7 M urea, 20 mM Sodium Acetate [5.2], 5 mM 2-mercaptoethanol, 1 mM EDTA [8.0], and approximately 10 g Dowex Marathon MR-3 hydrogen and hydroxide form) with 10 mL fractions at 5 mL/min for about 150 minutes. Fractions containing histone protein were pooled and put into Spectrum™ Spectra/Por™ 1 RC Dialysis Membrane Tubing 6-8 kDa MWCO (Thermo Fisher Scientific, 08-700-143) against cold deionized water (DI) containing 1 mM 2-mercaptoethanol at 4°C. DI was exchanged every 4 hours for 24 hours. After dialysis against DI, protein concentration was estimated by measuring absorption with extinction coefficient of each histone. If the concentration was lower than 2 mg/mL, protein solution was concentrated using a centrifugal device (PALL, PAMAP003c37). 2 mg of each purified histone was aliquoted in each tube. Subsequently, the tubes were frozen in liquid nitrogen and lyophilized. Lyophilized proteins were stored in -80°C.

*3-2. Histone octamer assembly and purification*

Each 2 mg of lyophilized core histone (H2A, H2B, H3, and H4) was dissolved in 1 mL unfolding buffer (6 M Guanidinium HCl and 20 mM Tris-HCl [7.5]) supplemented with 10 mM DTT and incubated for 1 hour. Each histone concentration was measured based on absorption, and equal molar amounts of histones were mixed. The mixture was dialyzed against Refolding buffer (2 M NaCl, 10 mM Tris-HCl [7.5], 1 mM EDTA, and 5 mM 2-mercaptoethanol) in Spectrum™ Spectra/Por™ 1 RC Dialysis Membrane Tubing 6-8 kDa MWCO (Thermo Fisher Scientific, 08-700-143) at 4°C overnight. The dialyzed sample was loaded onto HiLoad® 26/600 Superdex® 200 pg (Cytiva, GE28-9893-36) that was equilibrated with Refolding buffer. Proteins were eluted with 5 mL fractions at 2 mL/min, and all fractions were checked whether octamers were contained by running 18% SDS-PAGE gel. Histone octamer containing fractions were pooled and concentrated using a centrifugal device (PALL, PAMAP003c37). Histone octamers were stored at 4°C.

*3-3. In vitro nucleosome reconstitution*

*In vitro* nucleosome reconstitution was followed by the previous protocol (7). 500 mL of high-salt buffer (10 mM Tris-HCl [7.5], 1 mM EDTA, 2 M KCl, and 5 mM DTT), 2 L of low-salt buffer (10 mM Tris-HCl [7.5], 1 mM EDTA, 200 mM KCl, and 1 mM DTT), and 1 L of no salt buffer (10 mM Tris-HCl [7.5], 1 mM EDTA, and 1 mM DTT) were prepared and cooled at 4°C. 30~ 50 nM 200 bp Widom 601 DNA or 1~2 nM λ-DNA containing Widom 601 sequence was mixed with equi-volume of 4 M KCl to make the salt concentration equal to that of high-salt buffer (2 M). The DNA was mixed with purified histone octamers at approximately 1:1 molar ratio. High-salt buffer was added up to 40~50 μL. The mixture was dialyzed against high-salt buffer in a dialysis tube (Thermo Fisher Scientific, 69560) at 4°C. Then 1.5 L low-salt buffer was added to high-salt buffer with 1 mL/min rate by reducing the salt concentration from 2 M to 200 mM gradually during the dialysis. After 1.5 L of low-salt buffer was added, the dialysis buffer was changed to 500 mL of low-salt buffer for more than 4 hours. Then, the dialysis buffer was changed to 500 mL of no salt buffer for more than 4 hours. This step was repeated once again. Finally, the reconstituted nucleosomes were analyzed by gel shift assay. 10 nM of free DNA and nucleosomes were loaded on 6% non-denaturing PAGE in 0.5xTBE at 4°C for 50 minutes. The gel was imaged using a Typhoon RGB scanner (Cytiva).

*3-4. Micrococcal nuclease (MNase) assay*

To test nucleosome reconstitution *in vitro*, MNase assays were performed. 10 nM nucleosomes were incubated with 0.5 and 0.75 units/μl MNase (Enzynomics, M069S) at 37°C for 20 minutes. The reaction was stopped by 50 mM EDTA and 0.2 mg/ml Proteinase K followed by 30-minute incubation at 50°C. Digested DNA fragments were filtered by Expin™ PCR SV Kit (Gene All, 103-102) and then analyzed by 10% non-denaturing PAGE in 1x TBE at 23°C for 50 minutes. The gel was imaged using a Typhoon RGB scanner (Cytiva).

***4. DNA curtain assay***

*4-1. DNA curtain system*

For the DNA curtain experiments, total internal reflection fluorescence (TIRF) microscope combined with a fluidic system was used as previously described (8-11). For single-tethered DNA curtain experiments, a fused-silica slide with nano-trench patterns was assembled to a flow cell (12,13). The flow cells were washed sequentially with deionized water (DI) and lipid buffer (20 mM Tris-HCl [8.0], 100 mM NaCl). To form lipid bilayer on the slide surface, liposomes consisting of 0.5% biotinylated-DOPE (1,2-dioleoyl-sn-glycero-3-phosphoethanolamine-N-(cap biotinyl) (sodium salt)) (Avanti Polar Lipids, 870273), 8% mPEG 2000-DOPE (1,2-dioleoyl-sn-glycero-3-phosphoethanolamine-N-[methoxy(polyethylene glycol)-2000]) (Avanti Polar Lipids, 880130), and DOPC (1,2-dioleoyl-sn-glycero-3-phosphocholine) (Avanti Polar Lipids, 850375) were added to the flow cell. Lipid bilayer was promoted on the slide surface for 20 min after excess liposomes were removed by washing with lipid buffer. Only for double-tethered DNA curtain experiments, 0.1 mg/mL Anti-digoxigenin antibody (Roche, 11333089001) dissolved in lipid buffer was incubated for 20 minutes, which was nonspecifically adsorbed on pedestals. Then BSA buffer (40 mM Tris-HCl [8.0], 50 mM NaCl, 2 mM MgCl_2_, and 0.2 mg/mL BSA) was added for further surface passivation. Then the flow cells were incubated with 25 μg/mL streptavidin in BSA buffer for 10 min. After free streptavidin was washed out with BSA buffer, approximately 40 pM λ-DNA was added to the flow cell. Finally, the flow cell was equipped onto the TIRF microscope and connected with a fluidic system. All DNA curtain experiments were conducted at room temperature (23°C). DNA curtains were formed in Imaging buffer (Supplementary Table 3) at a flow rate of 0.5 mL/min.

Three lasers with different wavelengths (488 nm, 532 nm, and 637 nm) were used for the excitation of individual fluorophores. Each fluorescence was detected by an electron-multiplying camera (Andor Technology, iXon) through a proper optical filter set. All fluorescence images were recorded in NIS-Elements software (Nikon) and then analyzed by Image J (NIH).

*4-2. DNA curtain assay for Polθ_QM1*

Single-tethered DNA curtains were built up with λ-DNA containing the internal double-strand break with single-stranded overhang. 5 μM dATP, 20 nM Cy5-labeled oligomer with microhomology (Insert_polθ_3) and 14 nM Polθ_QM1 were introduced into the DNA curtains through a 100 μL loop at a flow rate of 0.5 mL/min. The mixture was incubated with λ-DNA for 5 minutes with the flow off. Then the flow was resumed, and unbound Cy5-labeled oligonucleotides were washed out. Fluorescence signals were collected using NIS-Elements software (Nikon).

*4-3. DNA curtain assay for Ku70/80*

To examine binding of Ku70/80 to blunt-ended DSBs, λ-DNA containing the Cy3-labeled triple internal DSBs was used for double-tethered DNA curtains. 5 nM Ku70/80 complexes were injected into the flow cell through a 100 μL loop at a flow rate of 0.2 mL/min. The Ku70/80 complexes were incubated for 5 minutes without flow. Then unbound Ku70/80 complexes were washed away by buffer flow. Subsequently, 20 nM Alexa647 FLAG antibody (Invitrogen, MA1-142-A647) was introduced into the flow cell and labeled to Ku70/80 complexes with 5-minute incubation. Excess antibodies were removed by buffer flow. Fluorescence signals were acquired using NIS-Elements software (Nikon).

*4-4. DNA curtain assay for APE1_D210N*

For fluorescent labeling of 3x HA-tagged APE1_D210N, HA-antibody (Invitrogen, 26183) was conjugated to 705 nm quantum dots (Qdots) using Qdot Antibody Conjugation Kit (SiteClick Qdot Antibody Labeling Kits, Thermo Fisher Scientific, S10454). APE1_D210N was incubated with HA-antibody conjugated Qdots at 1:3 molar ratio on ice for 10 minutes. Qdot-labeled APE1_D210N was diluted in imaging buffer (20 mM Tris-HCl [8.0], 0.1 mM MgCl_2_, 25 mM NaCl, 1 mM DTT, 1.6% glucose, 0.5x gloxy, and 0.05% BSA).

Double-tethered DNA curtains were formed using λ-DNA containing a single AP site, which was inserted based on our CRISPR-nCas9 system. To test binding of APE1_D210N on the λ-DNA, 50 pM Qdot-labeled APE1_D210N was injected into the flow cell. When maximum number of proteins arrived at DNA curtains, the flow was switched off. After 3-minute incubation, free Qdot-labeled proteins were washed out, and imaging was started in the absence of flow using NIS-Elements software (Nikon) at 10 frames per second. For the movement of APE1_D210N, immediately after Qdot-labeled APE1_D210N was injected, the flow was stopped, images were collected for 10 minutes.

***5. DNA curtain data analysis***

*5-1. Position distribution of fluorescence signal*

All the position distributions for fluorescent puncta (Cy3, Cy5, Cy5-labeled microhomology oligonucleotide (Insert_polθ_3), the Qdot-labeled APE1_D210N, and the Alexa 647–labeled Ku70/80) on λ-DNA were analyzed by fitting the fluorescence signals with a two-dimensional Gaussian function. The vertical center position of fluorescence puncta was considered as the binding position on λ-DNA. The binding distribution histogram was constructed with 1 kbp bin size. The error of each bar was determined by bootstrapping with 70% confidence interval.

*5-2. Calculation of diffusion coefficients of APE1_D210N*

The diffusion coefficients were calculated based on previous literature (13,14). Time traces of APE1_D210N were constructed using Particle tracker of Image J (NIH). 1D diffusion coefficients (*D*) were estimated by fitting of first three data points of MSD to prevent increase of the error of MSD. MSD was calculated based on mathematical definition given as

$$MSD \left( n, N \right)= \sum_{i=1}^{N-n} \frac{{(Y_{i+n}-Y_{i})}^{2}}{N-n}=2Dn\Delta t$$

where *N* is the total number of frames, *n* is the measurement window ranging from 1 to *N-1*, *Y* is the position of APE1_D210N along DNA, and Δt is the time interval between frames. The standard deviation (SD) of MSD, which was used for error, was calculated based on the equation,

$SD=\sqrt{\frac{\left( 2Di\Delta t \right)^{2}(2t^{2}+1)}{3i(N-i+1)}}$.

**6. Dual-bead optical trap assays**

Dual-trap optical trap assays were performed using a commercial C-Trap^®^ system (LUMICKS) equipped with a flow cell containing five microfluidic channels. Prior to sample introduction, all channels were equilibrated with 1x PBS buffer (137 mM NaCl, 2.7 mM KCl, 10 mM Na_2_HPO_4_, 1.8 mM KH₂PO₄). The protein channel was passivated with 20 mg/mL BSA in 1x PBS buffer. After passivation, the protein channel was filled with Ku70/80 buffer (40 mM Tris-HCl **[7.5]**, 50 mM KCl, 1 mM MgCl₂, 0.1 mg/mL BSA, 1 mM DTT, 500 µg/mL glucose oxidase, 40 µg/mL catalase, and 10% glucose). Both bead and DNA channels were washed with 1 mL of BSA buffer (40 mM Tris-HCl [8.0], 50 mM NaCl, 2 mM MgCl₂, and 0.2 mg/mL BSA).

The multiply-biotinylated λ-DNA was tethered between two 4.0–4.9 µm streptavidin-coated beads (11228, LUMICKS) in BSA buffer at a trap stiffness of 0.2–0.25 pN/nm. The presence of a single DNA molecule between the beads was confirmed using the worm-like chain model. For DSB tethering experiments, while λ-DNA containing an internal DSB was held at 10 pN in BSA buffer, Cy3 and Cy5 fluorescence signals were measured using 2D confocal scan mode. For Ku binding experiments, the λ-DNA was first tethered to the beads in BSA buffer and then moved into a protein channel containing 1 nM Ku70/80 and 3 nM Alexa647–labeled FLAG antibody (Invitrogen, MA1-142-A647) in Ku buffer (40 mM Tris-HCl [7.5], 50 mM KCl, 1 mM MgCl_2_, 0.1 mg/mL BSA, 1 mM DTT, 500 µg/mL glucose oxidase, 40 µg/mL catalase, and 10% glucose). Ku70/80 binding was monitored immediately afterward using kymograph mode.

**Supplementary Tables**

**Supplementary table 1. List of synthesized oligomers**

| **Oligomer name** | **Sequence (5’-3’)** | **Mfr.** |
| --- | --- | --- |
| 1036F | GCTACTGCAAGCTCTAGATTTCCATGTCGTCTGCCAG | Bionics |
| 1036R | GCTACTGCAAGCGCATGCTCGCTCGTAACTAAACAGGAG | Bionics |
| CY5-200 bp-F | [Cy5]CTGGATCCAGTCAGTACTG | Bionics |
| 200 bp-R | GTACTTAGGTCTCCATTGCTC | Bionics |
| Cy5-149 bp-F | [Cy5]AATCGGATGTATATATCTGACAC | Bionics |
| 149 bp-F | AATCGGATGTATATATCTGACAC | Bionics |
| Cy5-149 bp-R | [Cy5]CATCGAGAATCCCGG | Bionics |
| 149 bp-R | CATCGAGAATCCCGG | Bionics |
| Lambda biotin-L | [Phosphate]GGGCGGCGACCT[Biotin] | Bioneer |
| Lambda dig-R | [Phosphate]AGGTCGCCGCCC[Dig] | Bioneer |
| Lambda dig-L | [Phosphate]GGGCGGCGACCT[Dig] | Bioneer |
| Lambda biotin-R | [Phosphate]AGGTCGCCGCCC[Biotin] | Bioneer |
| IVT_Universal | AAAAAAGCACCGACTCGGTGCCACTTTTTCAAGTTGATAACGGACTAGCCTTATTTTAACTTGCTATTTCTAGCTCTAAAAC | Bionics |
| IVT_right_1 | GAAATTAATACGACTCACTATAGAGTGAAGCGTTATTGGTATGGTTTTAGAGCTAGAAATAGCAAG | Bionics |
| IVT_right_2 | GAAATTAATACGACTCACTATAGTATTCCTGATGTATCGATATGTTTTAGAGCTAGAAATAGCAAG | Bionics |
| IVT_middle_1 | GAAATTAATACGACTCACTATAGCACAGTCTGAGCGGTTCAACGTTTTAGAGCTAGAAATAGCAAG | Bionics |
| IVT_middle_2 | GAAATTAATACGACTCACTATAGAGTTCTGGCTGGAGTCAGTAGTTTTAGAGCTAGAAATAGCAAG | Bionics |
| IVT_left_1 | GAAATTAATACGACTCACTATAGATAAGAAAGCAGACGACATCGTTTTAGAGCTAGAAATAGCAAG | Bionics |
| IVT_left_2 | GAAATTAATACGACTCACTATAGTTACTGGGATGATCGTGAAAGTTTTAGAGCTAGAAATAGCAAG | Bionics |
| IVT_Widom_+3/+3.5_1 | GAAATTAATACGACTCACTATAGAGCGCGTACGTGCGTTTAAGGTTTTAGAGCTAGAAATAGCAAG | Bionics |
| IVT_Widom_+3/+3.5_2 | GAAATTAATACGACTCACTATAGGCTGTCTACGACCAATTGAGGTTTTAGAGCTAGAAATAGCAAG | Bionics |
| IVT_Widom_-6 | GAAATTAATACGACTCACTATAGGGGATTACTCCCTAGTCTCCGTTTTAGAGCTAGAAATAGCAAG | Bionics |
| Insert_right_Cy5 | [Cy5]TCGATACATCAGGAATATTTGATTCAGATGATATGACTATCAAGGCCGCCTGAGTGCGGTTTTACCGCAT | Bionics |
| Insert_middle_Cy5 | [Phosphate]TGACTCCAGCCAGAAC[Cy5-dT]GTTCATCCTTAAACCACTTGTGTGGGCATGAGCACCCGCGGCCCCTGTT | Bionics |
| Insert_AP | [Phosphate]TCGATACATCAGGAATATTTGATTCAGATGATA[dspacer]GACTATCAAGGCCGCCTGAGTGCGGTTTTACCGCAT | Bionics |
| Insert_DSB_1 | [CY5]TACTAACACGAAAGCTACGTCCAATTCTCGGCCTTGTGCATATATAACAT | Bionics |
| Insert_DSB_1_internal Cy5 | TACTAACACGAAAGCTACGTCCAA[CY5- dT]TCTCGGCCTTGTGCATATATAACAT | Bionics |
| Insert_DSB_2 | [Phosphate]GAACACGGACACGTTGGACTCGGAAGTTGTAGCAAATCCCACCCTGAACGTATCAAGGCCGCCTGAGTGCGGTTTTACCGCAT | Bionics |
| Insert_DSB_3 | [Phosphate]TCGATACATCAGGAATATTTGATTCAGATGATATGACCGTTCAGGGTGGGA[CY3-dT]TTGCT | Bionics |
| Insert_DSB_4 | [Phosphate]ACAACTTCCGAGTCCAACGTGTCCGTGTTCATGTTATATATGCACAAGGCCGAGAATTGGACGTAGCTTTCGTGTTAGTA | Bionics |
| Insert_TD_1A | TGAACCTATTCACTATTGGAGCATTCCGTTCGAGCATGGCAGTAAG[CY3-dT]ACGTTAACAAAATAATCATCCTTGCGCAGATTCCAGAT | Bionics |
| Insert_TD_1B | [Phosphate]CACGATCATCCCAGTAACGCGCCCGCGCTTCATCGCGTACTTACTGCCATGCTCGAACGGAATGCTCCAATAGTGAATAGGTTCA | Bionics |
| Insert_TD_2A | GTACTAAGACTTTCATGACTGCTCGACGTTTATGCTTTAGGCG[CY3-dT]GAAGAGTGTGTGGGCATGAGCACCCGCGGCCCCTGTT | Bionics |
| Insert_TD_2B | [Phosphate]TGACTCCAGCCAGAACTGTTCATCCTTAAACCACTCTCTTCACGCCTAAAGCATAAACGTCGAGCAGTCATGAAAGTCTTAGTAC | Bionics |
| Insert_TD_3A | GAACACGGACACGTTGGACTCGGAAGTTGTAGCAAATCCCACCCTGAACGTATCAAGGCCGCCTGAGTGCGGTTTTACCGCA | Bionics |
| Insert_TD_3B | [Phosphate]TCGATACATCAGGAATATTTGATTCAGATGATATGACCGT[CY3-dT]CAGGGTGGGATTTGCTACAACTTCCGAGTCCAACGTGTCCGTGTTC | Bionics |
| Insert_polθ_1 | ATCCCACCCTGAACGTATCAAGGCCGCCTGAGTGCGGTTTTACCGCAT | Bionics |
| Insert_polθ_2 | [Phosphate]TCGATACATCAGGAATATTTGATTCAGATGATATGACCGTTCAGGG[Cy3-dT]GGGATTTTTTTTTTTTTTTTAGACTC | Bionics |
| Insert_polθ_3 | [Cy5]TTTTTTTTTTTTTTTGAGTCT | Bionics |
| Insert_polθ_4 | TTTTTTTTTTTTTTTAGACTC | Bionics |
| Insert_Widom_fluorescein | [Phosphate]AATTGGTCGTAGACAGC[iFluorT]CTAGCACCGCTT | IDT |
| Insert_Widom_+3AP | [Phosphate]AATTGGTCGTAGACAGC[dSpacer]CTAGCACCGCTT | Bionics |
| Insert_Widom_+3.5AP | [Phosphate]AATTGGTCGTA[dSpacer]ACAGCTCTAGCACCGCTT | Bionics |
| Complementary_SHL+3/+3.5 | AAGCGGTGCTAGAGCTGTCTACGACCAATT | Bionics |
| Insert_Widom_-6AP | [Cy5]AATCGGATG[dSpacer]ATATATCTGACACGTGCCTGGA | Bionics |
| Complementary_SHL-6 | TCCAGGCACGTGTCAGATATATACATCCGATT | Bionics |
| Ku70/80_Cy5 | [Cy5]-TCGATTTAGCTCTGACGATTAGTCAGTAGTGAATTCGGATTACTGGGGCAGGGCAGGGCAGGGTCA | Bioneer |
| Ku70/80_comp | TGACCCTGCCCTGCCCTGCCCCAGTAATCCGAATTCACTACTGACTAATCGTCAGAGCTAAATCGA | Bioneer |
| AP_25_Cy5 | [Cy5]-GCGTCAAAATGT[dSpacer]GGTATTTCCATG | Bionics |
| AP_25_comp | CATGGAAATACCCACATTTTGACGC | Bionics |

**Supplementary table 2. List of sgRNAs and IVT primers**

| **sgRNA name** | **Used IVT primer** |
| --- | --- |
| sgRNA_right_1 | IVT_right_1, IVT_Universal |
| sgRNA_right_2 | IVT_right_2, IVT_Universal |
| sgRNA_middle_1 | IVT_middle_1, IVT_Universal |
| sgRNA_middle_2 | IVT_middle_2, IVT_Universal |
| sgRNA_left_1 | IVT_left_1, IVT_Universal |
| sgRNA_left_2 | IVT_left_2, IVT_Universal |
| sgRNA_Widom_+3/+3.5_1 | IVT_Widom_+3/+3.5_1, IVT_Universal |
| sgRNA_Widom_+3/+3.5_2 | IVT_Widom_+3/+3.5_2, IVT_Universal |
| sgRNA_Widom_-6 | IVT_Widom_-6, IVT_Universal |

**Supplementary table 3. Composition of imaging buffer**

| **Protein name** | **Imaging buffer composition** |
| --- | --- |
| Fluorophores | 40 mM Tris-HCl [8.0], 50 mM NaCl, 2 mM MgCl_2_, 0.2 mg/mL BSA, 0.1× gloxy, 3.6% glucose, and 0.1× Trolox |
| Ku70/80 | 40 mM Tris-HCl [7.5], 50 mM KCl, 0.1 mg/mL BSA, 5 mM MgCl_2_, 1 mM DTT, 0.2X gloxy, 1.6% glucose, 0.2X Trolox |
| Polθ_QM1 | 30 mM Tris-HCl [8.0], 6.4 mM NaCl, 1 mM DTT, 0.2X gloxy, 1.6% glucose, and 0.2× Trolox |
| APE1-D210N | 20 mM Tris-HCl [8.0], 0.1 mM MgCl_2_, 25 mM NaCl, 1 mM DTT, 1.6% glucose, 0.5x gloxy, and 0.05% BSA |

**Supplementary Figures**

**Figure S1.** **Preparation of nCas9 and single-guide RNA (sgRNA)**

A. SDS PAGE of purified nCas9 (D10A).

B. Schematic of sgRNA preparation.

C. PCR products of template DNAs for in vitro transcription (IVT) to produce sgRNAs.

D. 8 M urea denaturing gel for sgRNAs.

**Figure S2. APE1 purification and activity test**

A. SDS PAGE of purified wild-type APE1 and APE1_D210N.

B. Activity test for purified wild-type APE1 and APE1_D210N. 25 bp duplex DNA containing a single AP site is treated with either purified wild-type or APE1_D210N.

**Figure S3. Purification of Ku70/80 and Polθ and activity test**

A. Domain construct of (top) full-length Polθ and (bottom) Polθ_QM1, in which the amino acids (1~1791 aa) at the N-terminus are removed.

B. SDS PAGE of Polθ_QM1.

C. Activity test of purified Polθ_QM1 using bulk extension from microhomology.

D. SDS PAGE of Ku70/80. 6x His and 3x FLAG are tagged at the amino terminus of Ku80.

E. EMSA of Ku70/80. 5 nM Cy5-labeled 66 bp duplex DNA is incubated with Ku70/80 at different concentrations (0, 1, 3, 5, 10, 30, 50, and 100 nM).

F. Supershift of DNA bound Ku70/80 by anti-FLAG antibodies. 5 nM Cy5-labeled duplex DNA is pre-incubated with 15 nM Ku70/80. Then anti-FLAG antibody is titrated at different concentrations (3, 30, and 300 nM).

G. Kymograph of Ku binding to the internal DSB using the dual-trap optical tweezers combined with the confocal fluorescence imaging. Green signal represents Cy3 fluorescence indicating the internal DSB. Ku70/80 labeled with Alexa647-antibody appears and stably stays at the same position as Cy3, indicating that Ku70/80 binds the internal DSB through 3D collision.

**Figure S4. Position change of a Cy5-labeled oligonucleotide insertion in λ-DNA**

A. (Top) Schematic of λ-DNA containing a Cy5-labeled oligonucleotide at 21,647 bp. (Bottom) Schematic of DNA curtain with λ-DNA containing Cy5-labeled oligonucleotide at 21,647 bp.

B. DNA curtain image for λ-DNA containing Cy5-labeled oligonucleotide at 21,647 bp in the (top) presence and (bottom) absence of buffer flow. Cy5 insertion position is denoted by a magenta arrowhead at the left. “B” at the left and black arrow at the right indicate barrier position and flow direction, respectively.

C. Position distribution of Cy5 fluorescence puncta. The center of the magenta peak is 21.5 ± 1.5 kbp from fitting with a single Gaussian function and well matches with a Cy5 insertion position. The weak pink peak is adopted from Figure 1G.

**Figure S5. Insertion of a DNA double-strand break (DSB).**

A. Schematic of Cy3-labeled DSB with a single-stranded overhang around 33.5 kbp.

B. (Top) Image for single-tethered DNA curtains with a Cy3-labeled DSB, which is inserted into around 15 kbp. (Bottom) Biotin is labeled at the opposite end of λ-DNA and hence the DSB is placed around 33.5 kbp. “B” at the left and black arrow at the right represent the barrier position and flow direction, respectively. The DSB position is indicated by a green arrowhead.

C. Position distribution of Cy3 fluorescent puncta in Figure S5B. λ-DNA constructs are shown above with DSB position. The distinct peak is fitted with a single Gaussian function (red line). The peak center is 14.8 ± 1.5 kbp or 33.8 ± 1.8 kbp, which well matches the DSB position. N indicates total number of analyzed molecules. Error bars are obtained from bootstrapping with 70% confidence intervals.

D. Schematic of dual-bead optical tweezers combined with confocal fluorescence imaging (Lumicks, C-trap). λ-DNA having a blunt-end DSB at ~ 33.5 kbp is hung between two optically trapped beads. The blunt-end DSB is labeled with Cy3 and Cy5.

E. A representative kymograph of Cy3 and Cy5 fluorescence signals.

F. Relative fraction of Cy3 and/or Cy5 fluorescence signals. Total number of molecules analyzed is 19. Error bars are obtained from 95% confidence intervals using the Clopper-Pearson binomial methods.

**Figure S6. SMARCA4 effect on APE1 cleavage of the AP site in a nucleosome**

A. Denaturing gel images showing APE1-mediated cleavage of AP sites in naked DNA at different AP-site positions.

B. Quantification of the data shown in Figure S6A. Errors are standard errors in triplicate.

C. Activity test of recombinant SMARCA4. SMARCA4 repositions histone octamers in the presence of ATP.

D. APE1 cleavage of the AP site at SHL +3 on bare DNA (200 bp Widom 601), nucleosomes, and nucleosomes incubated with 3 nM SMARCA4.

E. Quantification of the data shown in Figure S6D. Errors are standard errors in triplicate.

**Supplementary references**

1.	Lee, J., Lim, K., Kim, A., Mok, Y.G., Chung, E., Cho, S.-I., Lee, J.M. and Kim, J.-S. (2023) Prime editing with genuine Cas9 nickases minimizes unwanted indels. *Nature Communications*, **14**, 1786.

2.	Hanakahi, L.A. (2007) 2-Step purification of the Ku DNA repair protein expressed in Escherichia coli. *Protein expression and purification*, **52**, 139-145.

3.	Hogg, M., Seki, M., Wood, R.D., Doublié, S. and Wallace, S.S. (2011) Lesion bypass activity of DNA polymerase θ (POLQ) is an intrinsic property of the pol domain and depends on unique sequence inserts. *Journal of molecular biology*, **405**, 642-652.

4.	Tomida, J., Takata, K.-i., Lange, S.S., Schibler, A.C., Yousefzadeh, M.J., Bhetawal, S., Dent, S.Y. and Wood, R.D. (2015) REV7 is essential for DNA damage tolerance via two REV3L binding sites in mammalian DNA polymerase ζ. *Nucleic acids research*, **43**, 1000-1011.

5.	Zahn, K.E., Averill, A.M., Aller, P., Wood, R.D. and Doublié, S. (2015) Human DNA polymerase θ grasps the primer terminus to mediate DNA repair. *Nature structural & molecular biology*, **22**, 304-311.

6.	Luger, K., Rechsteiner, T.J. and Richmond, T.J. (1999) Expression and purification of recombinant histones and nucleosome reconstitution. *Methods Mol Biol*, **119**, 1-16.

7.	Mattiroli, F., Gu, Y. and Luger, K. (2018) Measuring Nucleosome Assembly Activity in vitro with the Nucleosome Assembly and Quantification (NAQ) Assay. *Bio Protoc*, **8**.

8.	Kang, Y., An, S., Min, D. and Lee, J.Y. (2022) Single-molecule fluorescence imaging techniques reveal molecular mechanisms underlying deoxyribonucleic acid damage repair. *Frontiers in Bioengineering and Biotechnology*, **10**, 973314.

9.	Kim, S., Kim, Y. and Lee, J.Y. (2024) Real-time single-molecule visualization using DNA curtains reveals the molecular mechanisms underlying DNA repair pathways. *DNA repair*, **133**, 103612.

10.	Kang, Y., Bae, S., An, S. and Lee, J.Y. (2022) Deciphering molecular mechanism of histone assembly by DNA curtain technique. *JoVE (Journal of Visualized Experiments)*, e63501.

11.	Cha, J. and Lee, J.Y. (2021) A novel high-throughput single-molecule technique: DNA curtain. *Journal of the Korean Physical Society*, **78**, 442-448.

12.	Kang, Y., Cheon, N.Y., Cha, J., Kim, A., Kim, H.i., Lee, L., Kim, K.O., Jo, K. and Lee, J.Y. (2020) High‐throughput single‐molecule imaging system using nanofabricated trenches and fluorescent DNA‐binding proteins. *Biotechnology and Bioengineering*, **117**, 1640-1648.

13.	Cheon, N.Y., Kim, H.-S., Yeo, J.-E., Schärer, O.D. and Lee, J.Y. (2019) Single-molecule visualization reveals the damage search mechanism for the human NER protein XPC-RAD23B. *Nucleic acids research*, **47**, 8337-8347.

14.	An, S., Kusakabe, M., Kim, H.-S., Kozono, H., Cheon, N.Y., Kim, J., Kang, J., Jang, S., Sugasawa, K. and Schärer, O.D. (2025) XPC-RAD23B enhances UV-DDB binding to DNA to facilitate lesion search in nucleotide excision repair. *Nucleic Acids Research*, **53**, gkaf463.
